# Molecular origin of AuNPs-induced cytotoxicity and mechanistic study

**DOI:** 10.1038/s41598-019-39579-3

**Published:** 2019-02-21

**Authors:** Euiyeon Lee, Hyunjin Jeon, Minhyeong Lee, Jeahee Ryu, Chungwon Kang, Soyoun Kim, Junghyun Jung, Youngeun Kwon

**Affiliations:** 1 Department of Biomedical Engineering (BK21 plus), Dongguk University, Pildong 3-ga, Seoul, 04620 Korea; 2 Department of Life Science, Dongguk University, Pildong 3-ga, Seoul, 04620 Korea

## Abstract

Gold nanoparticles (AuNPs) with diverse physicochemical properties are reported to affect biological systems differently, but the relationship between the physicochemical properties of AuNPs and their biological effects is not clearly understood. Here, we aimed to elucidate the molecular origins of AuNP-induced cytotoxicity and their mechanisms, focusing on the surface charge and structural properties of modified AuNPs. We prepared a library of well-tailored AuNPs exhibiting various functional groups and surface charges. Through this work, we revealed that the direction or the magnitude of surface charge is not an exclusive factor that determines the cytotoxicity of AuNPs. We, instead, suggested that toxic AuNPs share a common structural characteristics of a hydrophobic moiety neighbouring the positive charge, which can induce lytic interaction with plasma membrane. Mechanistic study showed that the toxic AuNPs interfered with the formation of cytoskeletal structure to slow cell migration, inhibited DNA replication and caused DNA damage via oxidative stress to hinder cell proliferation. Gene expression analysis showed that the toxic AuNPs down-regulated genes associated with cell cycle processes. We discovered structural characteristics that define the cytotoxic AuNPs and suggested the mechanisms of their cytotoxicity. These findings will help us to understand and to predict the biological effects of modified AuNPs based on their physicochemical properties.

## Introduction

The biomedical applications of nanotechnology have been expanding rapidly during last decades. Among various metal nanoparticles, gold nanoparticles (AuNPs) have attracted special interests for sensing^1,2^, bio-imaging^3–6^ and drug delivery^3,7–9^, owing to their tunability and biocompatibility as well as unique optical properties. Despite the large potential in biomedical applications, *in vivo* usage of AuNPs is still limited mainly due to the shortage of understanding on how AuNPs interact and affect biological systems.

It is generally agreed that the biological effects of AuNPs are directly influenced by their physicochemical properties such as size, shape, charge, surface functional groups as well as aggregation states^10–17^. However, the rules governing the molecular interactions of AuNPs with their target cells remain largely unexplored. The ionic interactions between the plasma membrane and the AuNPs, determined by the surface charge of AuNPs, were suggested as one such mechanism of action^18,19^. These interactions could, in turn, determine intracellular uptake of AuNPs and their biological effects. While a large number of scientific reports specifically addressed the cytotoxicity of AuNPs in association with their surface charge, the reported results are somewhat conflicting^10,20–26^. On one hand, several research groups suggested that AuNPs are not cytotoxic regardless of their surface charge. For example, Connor *et al*. reported variously charged AuNPs did not show noticeable toxicity to a human leukemia cell line K562^21^. Li *et al*. tested cell viability with AuNPs modified with anionic, cationic and neutral functional groups to show that all modified AuNPs had no effect on viability of human bone marrow-derived mesenchymal stem cells^23^. Shukla *et al*. also showed that lysine and poly(L-lysine) conjugated cationic AuNPs were not cytotoxic and, furthermore, that the amount of reactive oxygen species inside the cells was reduced by lysine-AuNPs^26^. On the other hands, many research groups reported cytotoxic effects of cationic AuNPs. Goodman *et al*. reported that the surface charge of the nanoparticle plays a key role in determining toxicity, showing that cationic AuNPs displaying moderate toxicity while their anionic counterparts exhibit no toxic effects^10^. Fiqueroa *et al*. tested cytotoxicity of various AuNPs-poly(amidoamine) (PAMAM) conjugates using human breast adenocarcinoma cells (SkBr3). They reported the cytotoxicity increased as the number of PAMAM dendrimers bound to AuNPs increased^22^. Chauhan *et al*. also suggested that the high density of primary amine groups increased the toxicity of dendrimers^20^. Alternatively, Schaeublin *et al*. argued both positively and negatively charged AuNPs are cytotoxic, with the negatively charged particles evoking greater responses^25^. Despite the relative wealth of toxicity studies focusing on charged AuNPs, contradictory results remain as the main obstacle in transitioning nanotechnology into the clinical settings. Therefore it is important to study nanoparticle toxicity more systematically using well-tailored AuNPs, in which we can fine-tune the physicochemical properties to study their effects.

In this paper, we carried out a systematic study using a library of well-dispersed AuNPs presenting a variety of surface functional groups with a spectrum of charges ranging from −42.8 ± 11.8 to +41.8 ± 3.8 mV. Special interests were paid to cationic AuNPs as they are often mentioned as attractive platforms for drug delivery vehicles with considerable controversies on their toxicity. We attempted to explain the origin of toxicity in relation to the magnitude of charge, surface functional groups, and the ligand structures. We also tried to understand the mechanistic aspect of AuNPs-induced cytotoxicity by looking at various cell functions as well as by gene expression profiling. The findings of this work may reduce the serious controversies concerning the toxicological effects of modified AuNPs and facilitate the biomedical applications of nanotechnology.

## Results

### Generation of a charge library of modified AuNPs

**Figure 1:**
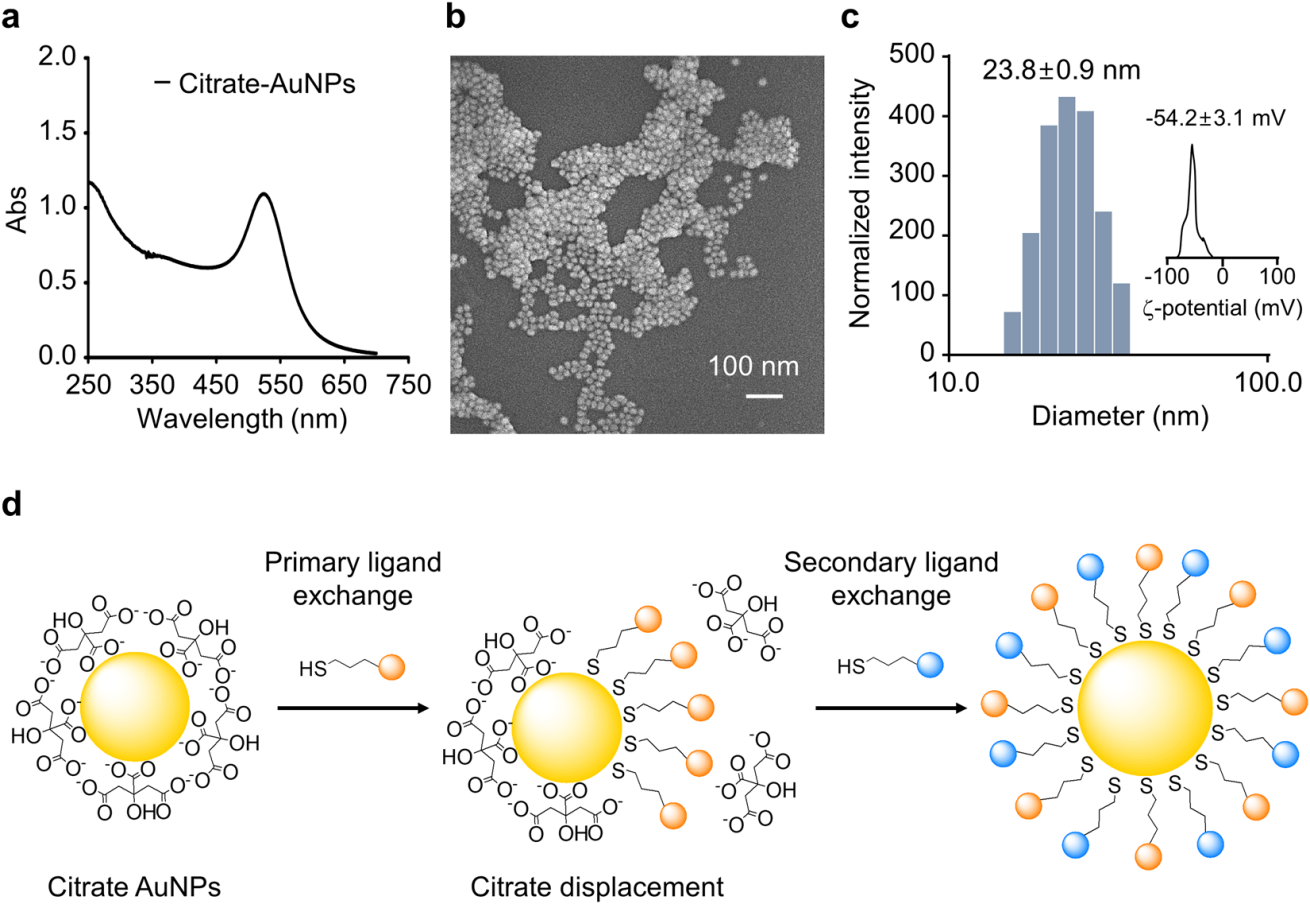
Characterization of gold nanoparticles (AuNPs) and schematics of AuNP modification. AuNPs were synthesized and analysed using UV-Vis spectroscopy (**a**), Field emission scanning electron microscopy (FE-SEM) at 5 kV (**b**), Dynamic laser scattering (DLS) (**c**), and zeta-potential measurements (**c**, inset). AuNPs were modified via place-exchange reaction to introduce variously charged ligands (**d**).

In order to investigate the effect of charged AuNPs on biological systems, we first prepared a series of well-dispersed AuNPs with different surface charges and functional groups by modifying AuNPs with various thiol ligands. We prepared 20 nm-diameter citrate-capped gold nanospheres as they are reasonably stable during long term storage and relatively safe for biomedical applications^27,28^ (Fig. 1a–c). The thiol ligands were either commercially available organic thiols or Cys-containing synthetic peptides (Fig. S1). Peptide ligands were chosen because they are popular as targeting ligands in biomedical applications^29,30^. They are also appropriate for the introduction of various functional groups of different charges with structural diversity. The amino acid sequences were selected to make anionic, neutral, and cationic peptide-ligands based on their pI values (Table 1).

**Table 1:** Size, zeta(ζ)-potential, and UV-Vis spectra of modified gold nanoparticles (AuNPs) in deionized (DI) water. Hydrodynamic radii, ζ-potential values are represented as means ± standard deviation (n = 3) for all AuNPs.

Sample	Ligand structure	pI value	Hydrodynamic raddi (nm)	Polydispersity index (PDI)	ζ-potential (mV)	λ-max (nm)
Mesna-AuNPs	NaSO_3_CH_2_CH_2_SH	—	32.8 ± 0.5	0.246	−42.8 ± 11.8	524
AP1-AuNPs	Acetyl-Asp-Asp-Asp-Tyr-Cys	3.6	36.6 ± 0.9	0.171	−22.3 ± 0.5	530
AP2-AuNPAPs	Acetyl-Glu-Glu-Glu-Gly-Tyr-Cys	3.8	25.2 ± 0.3	0.243	−20.4 ± 1.2	525
AP3-AuNPs	Acetyl-Asp-Asp-Asp-Gly-Tyr-Cys	3.6	24.8 ± 0.3	0.195	−13.3 ± 0.4	529
mPEG_350_-AuNPs	CH_3_O(CH_2_CH_2_O)_n_CH_2_CH_2_SH	—	29.3 ± 0.9	0.210	−3.2 ± 5.9	527
NP1-AuNPs	Asp-Asp-Asp-Tyr-Cys	3.3	30.4 ± 1.4	0.167	4.6 ± 0.5	530
NP2-AuNPs	Ala-Ala-Ala-Gly-Tyr-Cys	5.3	24.9 ± 0.5	0.256	−5.2 ± 0.6	527
NP3-AuNPs	Acetyl-Ser-Ser-Ser-Gly-Tyr-Cys	7.0	24.7 ± 0.5	0.256	−7.2 ± 1.1	527
MUAM-AuNPs	NH_2_CH_2_(CH_2_)_9_CH_2_SH	—	49.1 ± 1.3	0.226	41.8 ± 3.8	522
CP1-AuNPs	Arg-Arg-Arg-Gly-Tyr-Cys	11.1	45.1 ± 0.4	0.280	31.4 ± 1.5	526
CP2-AuNPs	Lys-Lys-Lys-Gly-Tyr-Cys	10.1	38.7 ± 0.4	0.266	27.8 ± 3.8	532
CP3-AuNPs	Arg-Gly-Tyr-Cys	8.6	40.6 ± 1.4	0.251	24.1 ± 2.0	524
CP4-AuNPs	Lys-Gly-Tyr-Cys	8.6	32.8 ± 1.4	0.242	22.3 ± 1.0	533
CP1M1-AuNPs	Arg-Arg-Arg-Gly-Tyr-Ahx-Cys	11.1	48.1 ± 0.1	0.256	22.1 ± 0.6	533
CP1M2-AuNPs	Arg-Arg-Arg-Gly-Tyr-Lys-C_11_-Cys	11.4	35.0 ± 0.6	0.276	26.7 ± 2.1	526

Initially, direct ligand exchange was attempted to generate modified AuNPs. At neutral pH, the citrates were easily replaced with neutral or anionic thiol ligands. We, however, observed considerable aggregation when cationic thiol ligands were added to citrate-capped AuNPs (Fig. S2). It was consistent with previous reports that the introduction of cationic ligands in the presence of citrates often cause aggregation due to the ionic interaction^31–33^. In order to overcome this problem, we adopted the ‘place-exchange reaction’ with necessary alterations (Fig. 1d). Briefly, the surfactant citrates were first replaced with neutral primary thiol ligand, i.e., methoxy-polyethyleneglycol thiol (mPEG-SH; average molecular weight of 350 Da). The surfactant solution was removed from the AuNPs and the secondary ligands was then added to the AuNPs. The surface properties of the modified AuNPs were mainly determined by the nature of the secondary ligands introduced. This approach provided a reliable protocol to fabricate well-dispersed AuNPs of various surface charges.

The modified AuNPs were characterized by UV-Vis spectroscopy, Dynamic laser scattering (DLS) measurement, and Zeta (ζ)-potential measurement before we evaluate their biological effects (Table 1 and Fig. S3). UV-Vis spectra showed that the absorption maximum of AuNPs shifted from 520 to 522 ~ 533 nm upon the introduction of thiol ligands. None of the modified AuNPs showed severe spectral changes, i.e. the sign of aggregation. A new absorption maximum at 280 nm was also observed when a tyrosine-containing ligand was introduced. The hydrodynamic radii of modified AuNPs increased slightly with a narrow size distribution. The measured sizes were between 24.7 ± 0.5 to 49.1 ± 1.3 nm with poly dispersity index (PDI) smaller than 0.3 suggesting that the particles are well dispersed. The surface charges of the modified AuNPs were the most negative for sodium 2-mercaptoethane sulfonate (mesna)-AuNPs at −42.8 ± 11.8 mV and the most positive for 11-mercaptoundecylamine (MUAM)-AuNPs at 41.8 ± 3.8 mV. The charge library of AuNPs includes Anionic peptide 1 (AP1)-AuNP (ζ-potential = −22.3 ± 0.5 mV), AP2-AuNP (−20.4 ± 1.2 mV), AP3-AuNP (−13.3 ± 0.4 mV), mPEG_350_-AuNPs (−3.2 ± 5.9 mV), Neutral peptide 1 (NP1)-AuNP (4.6 ± 0.5 mV), NP2-AuNP (−5.2 ± 0.6 mV), NP3-AuNP (−7.2 ± 1.1 mV), Cationic peptide 1 (CP1)-AuNPs (31.4 ± 1.5 mV), CP2-AuNPs (27.8 ± 3.8 mV), CP3-AuNPs (24.1 ± 2.0 mV), CP4-AuNPs (22.3 ± 1.0 mV), modified CP1-1 (CP1M1)-AuNPs (22.1 ± 0.6 mV), CP1M2-AuNPs (26.7 ± 2.1 mV) (Table 1). The cationic AuNPs showed a positive correlation between the magnitude of the charge and the size of AuNPs. This is likely because the higher magnitude of surface charge results in the further extended Stern double layer and, consequently, the increase of hydrodynamic radius^34,35^. The UV-Vis spectra, DLS, and ζ-potential measurement together confirmed that we prepared a library of well-dispersed AuNPs with varying charges.

### Effect of modified AuNPs on cell viability

In order to monitor the effect of modified AuNPs on the viability of cells, two different viability assays, MTT and trypan blue assay, were carried out. We used two different assays because the MTT assay reports the number of live cells by monitoring mitochondrial activity while the trypan blue assay reports the number of dead cells with compromised plasma membranes. MTT assay was performed on HeLa and Normal human dermal fibroblast (NHDF) cells and trypan blue assays were carried out on HeLa cells. HeLa cells were chosen because there are a large pool of toxicity test data available for comparison^36–39^. Cells in the logarithmic growth phase were used as they are generally more vulnerable to toxicants.

**Figure 2:**
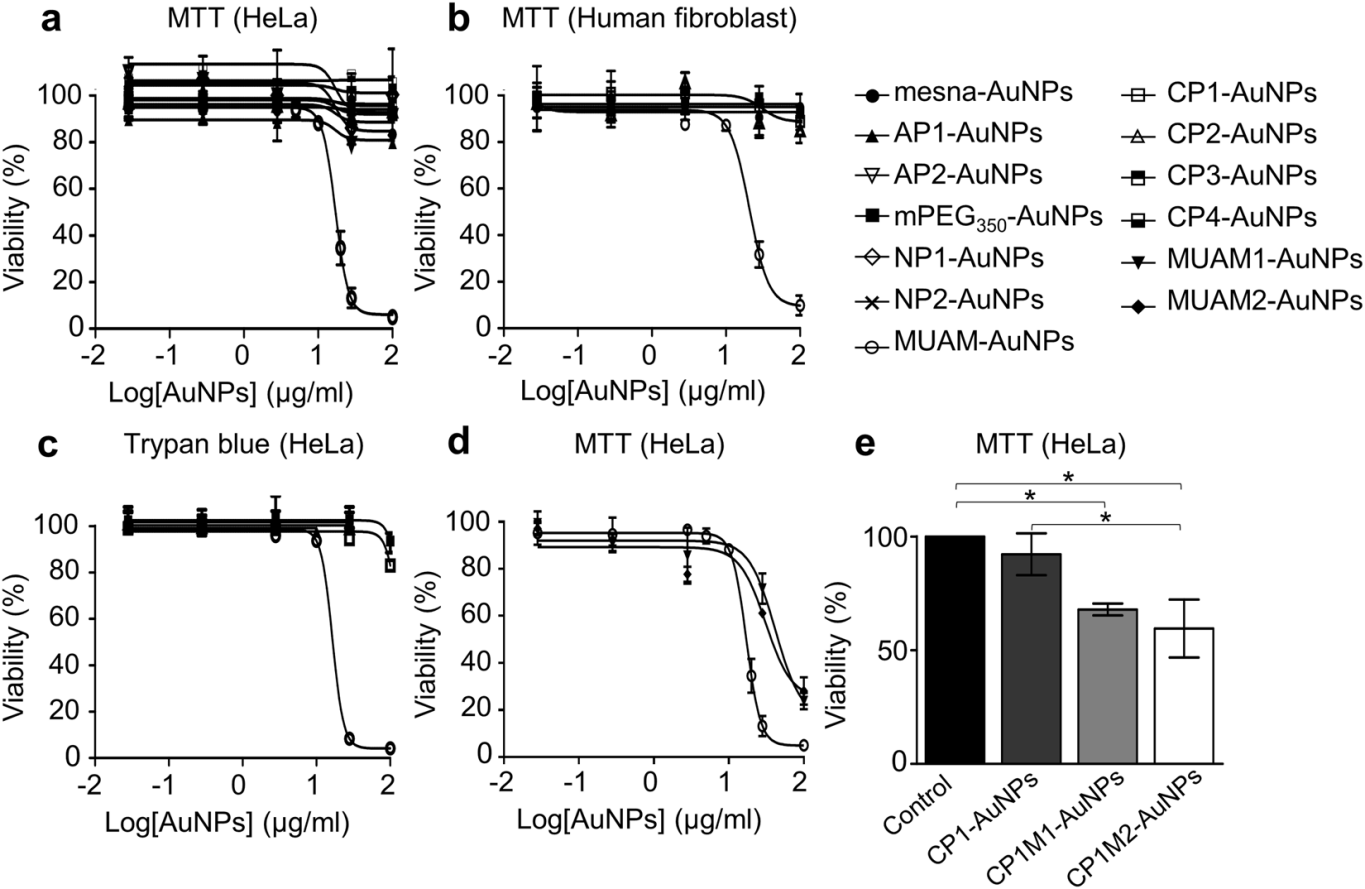
Effect of modified gold nanoparticles (AuNPs) on cell viability. (**a**) The viability of AuNPs-treated HeLa cells were analysed using MTT assays. Only MUAM-AuNPs induced cell death with LD_50_ of 17.1 μg/ml. (**b**) The viability of AuNPs-treated human fibroblasts were analysed using MTT assays. Only MUAM-AuNPs induced cell death with LD_50_ of 20.5 μg/ml. (**c**) A trypan blue assay was performed on HeLa cells treated with modified AuNPs for 24 h. Only MUAM-AuNPs induced cell death with LD_50_ of 16.5 μg/ml. (**d**) MTT viability assay was performed on cells treated with three different MUAM-carrying AuNPs (MUAM-, MUAM1- and MUAM2-AuNPs). All three AuNPs showed comparable cytotoxicity regardless of ligand densities or the magnitude of positive charges. (**e**) MTT viability assay was performed on Cells treated with three different CP1-derived AuNPs (CP1-, CP1M1- and CP1M2-AuNPs). The introduction of hydrophobic chains increased cytotoxicity of CP1-derived AuNPs. The results are shown as mean ± standard error of mean (*p < *0.05*, one-way ANOVA).

The MTT assay showed that only MUAM-AuNPs, among 15 different modified AuNPs, were significantly cytotoxic for both cell lines (Fig. 2a,b). The median lethal dose (LD_50_)’s of MUAM-AuNPs were 17.1 μg/ml for HeLa and 20.5 μg/ml for NHDF. AuNPs modified with other ligands did not show measurable cytotoxicity at concentrations up to 100 μg/ml. The toxicities of free ligands were also measured in order to eliminate the ligand effect on cell viability. The result showed the free ligand were not toxic excluding the influence of trace amount of free ligand detached from AuNPs as the cause of cytotoxicity (Fig. S4). Trypan blue assay also showed that only MUAM-AuNPs were cytotoxic among all the modified AuNPs tested. The determined LD_50_ of MUAM-AuNPs was 16.5 μg/ml, which is comparable to the result from the MTT assay (Fig. 2c). These two assays confirmed that cationic MUAM-AuNPs showed acute toxicity and raised two questions. Would these modified AuNPs affect the functions of cells at below lethal concentration, which could lead to chronic or long-term toxicity? Why only MUAM-AuNPs are cytotoxic among several cationic AuNPs?

### Effects of modified AuNPs on cellular functions

In studying nanotoxicology, not only the acute toxicity but also secondary or long term toxicity need to be considered^40,41^ because AuNPs can cause impairment in cell functions to trigger abnormal cell/tissue development at below lethal concentrations. We therefore studied the effect of AuNPs on cellular functions by monitoring cell motility, proliferation, DNA replication, and DNA damage. Cells were treated with modified AuNPs at 10 μg/ml as it is the highest concentration that did not alter the cell viability, lethal dose 0% (LD_0_). We also observed the changes in the cytoskeletal structures and ROS concentrations. For the damaged functions, we tried to determine whether the inhibition was via direct interaction or via indirect disruption of related signalling pathways. We chose mesna-AuNPs, mPEG_350_-AuNPs, MUAM-AuNPs, CP1-AuNPs, and CP2-AuNPs as representative modified AuNPs to be tested.

#### Effect of AuNPs on cell motility and cytoskeletal structures

**Figure 3:**
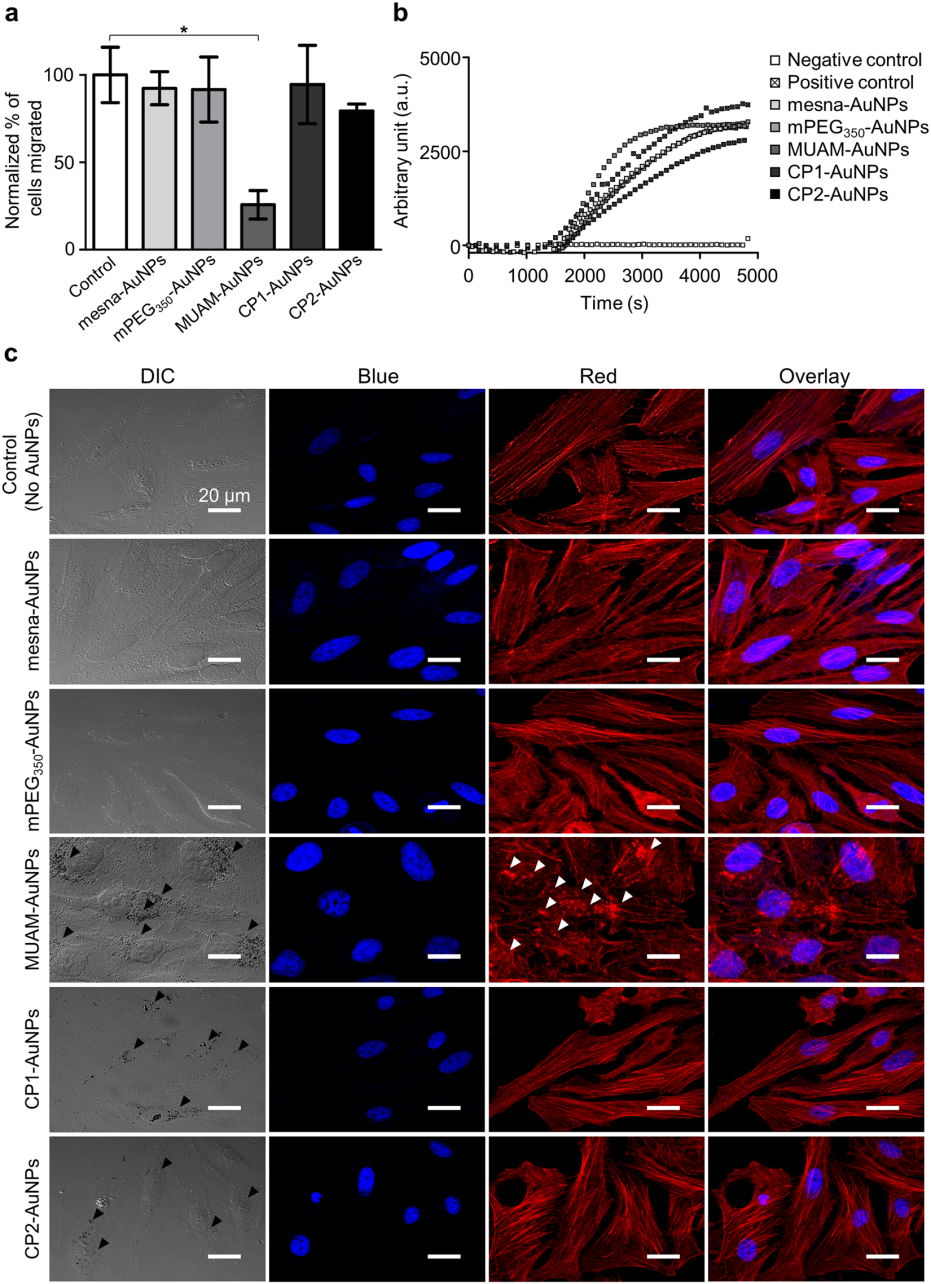
Effect of modified gold nanoparticles (AuNPs) on cell motility. (**a**) The motility of AuNPs-treated cells were monitored via a gap-filling assay. The migration rate decreased when cells were treated with MUAM-AuNPs (10 μg/ml) (*p < *0.05*, one-way ANOVA). (**b**) *In vitro* actin polymerization assay was performed on HeLa cells treated with modified AuNPs (10 μg/ml). The rate of actin polymerization did not change noticeably when treated with AuNPs. (**c**) Cytoskeletal structures in AuNPs-treated cells were visualized using fluorescent phalloidin (DAPI-stained nucleus, blue; actin filaments, red). F-actins in MUAM-AuNPs treated cells were disassembled and fragmented (white arrows). Scale bar: 50 μm.

Cell migration is a crucial process for the survival and differentiation of mammalian cells^42,43^. Various external signals control the motility of cells and a number of diseases are influenced by inappropriate regulations of cell migration^44,45^. We first studied the effect of AuNPs on the motility of HeLa cells via a gap-filling assay. The number of migrated cells were counted and normalized using the solvent-treated negative control cells. The motility decreased by 70% when the cells were treated with MUAM-AuNPs (Fig. 3a). CP2-AuNPs also reduced cell motility slightly but not with statistical significance (*p* > *0.1*, one-way ANOVA). Cells treated with other AuNPs did not show noticeable change.

As an attempt to explain the retarded migration, we looked into the changes in cytoskeletal structure of AuNPs-treated cells by staining F-actins using fluorescent Phalloidin. MUAM-AuNPs treated cells lost well-organized cytoskeletal structures exposing disassembled and fragmented F-actins (white arrows) with more rounded morphology, while other AuNPs-treated cells maintained stretch long F-actin fibres (Fig. 3c). The loss of long F-actins could explain the decreased motility of MUAM-AuNPs treated cells, because F-actins align with the migration axis to facilitate the movement^46^. The changes in cytoskeletal structure could also disrupt the extracellular matrix organization to result in slower migration^36^.

We then carried out *in vitro* actin polymerization assay in the presence of AuNPs in order to answer whether MUAM-AuNPs interfere with actin polymerization in direct manner or indirectly. The actin filaments formed in the presence of MUAM-AuNPs were shorter and more nucleated compared with the untreated control (Fig. S5). Other AuNPs did not make noticeable differences. While MUAM-AuNPs altered the shape of the F-actins, the rates of polymerization were comparable between all tested samples (Fig. 3b). The results suggest that MUAM-AuNPs act as a severing agent on actin filaments to make fragmented and nucleated F-actins rather than inhibiting the polymerization^47,48^. These *in vitro* actin polymerization studies suggest that MUAM-AuNPs alter the cytoskeletal structure by directly interfering with F-actin formation rather than tweaking the migration related signalling pathways.

#### Effect of AuNPs on cell division and proliferation

**Figure 4:**
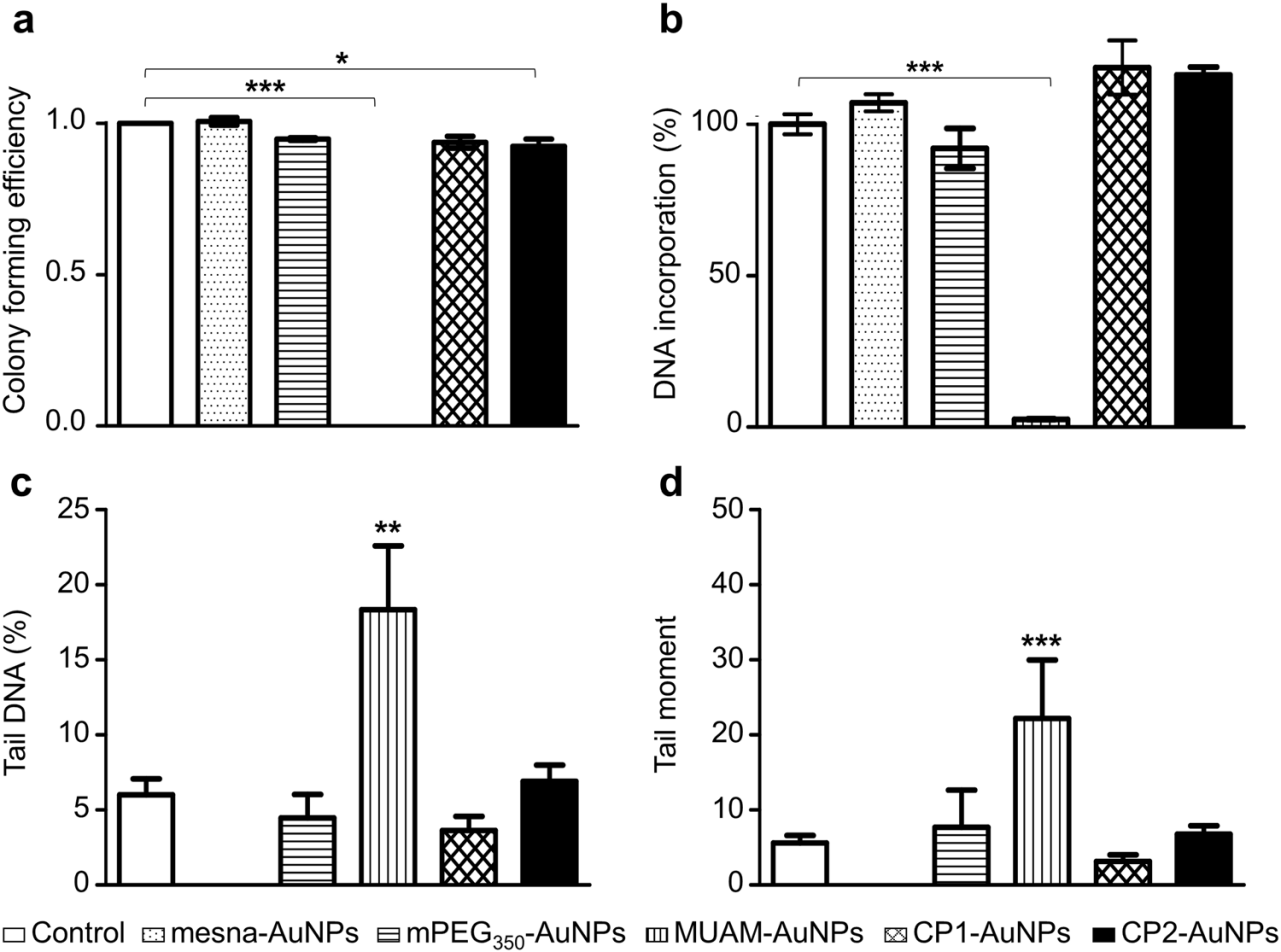
Effect of modified gold nanoparticles (AuNPs) on cell division and proliferation. (**a**) The Colony forming efficiency assay was performed on cell treated with modified AuNPs (10 μg/ml). MUAM-AuNPs treated cells did not form colonies over 50 cells. The number of colonies also decreased slightly in CP2-AuNP treated cells. (**b**) The effect of modified AuNPs on DNA replication was analysed. MUAM-AuNPs inhibited DNA replication near to completion. (c and d) AuNPs-induced DNA damage was monitored using the Comet Assay. Measurements of % tail DNA (**c**) and tail moment (**d**) suggest severe DNA damage in MUAM-AuNPs treated samples (*p < *0.05*, **p < *0.01*, ***p < *0.001*, one-way ANOVA).

We next studied whether cell division and proliferation related checkpoints are well functioning in the presence of modified AuNPs via colony forming efficiency (CFE) assay. The cells treated with neutral or anionic AuNPs did not show a notable difference compared with the control (Fig. 4a). CP1-AuNPs and CP2-AuNPs treated cells also showed comparable numbers of colonies. Conversely, MUAM-AuNPs treated cells showed a striking difference by producing no colonies of over 50 cells, while there still were viable cells observed. This result states that proliferation-related cell functions were severely damaged by treating with MUAM-AuNPs at LD_0_.

There could be multiple factors leading to the suppressed proliferation. For example, above-mentioned cytoskeletal structure disruption by MUAM-AuNPs can repress cell division by impeding cell polarization^49^ or by impairing the crosstalk between actin cytoskeleton and β-tubulins to disable mitosis^50,51^. The inhibition of DNA replication and DNA damage could be other causes^52^. We first looked into DNA replication using BrdU incorporation assay. The quantitative analysis of newly synthesized DNA indicates that MUAM-AuNPs inhibited DNA replication near to completion, while other cationic AuNPs, CP1-AuNPs and CP2-AuNPs, did not make noticeable changes compared with the untreated control (Fig. 4b). We also noticed AuNPs do not inhibit *in vitro* DNA polymerization at LD_0_. The result may suggest that the inhibited DNA replication is not due to the direct interaction between AuNPs and DNA or polymerase, but more likely due to the complicated interference on signalling pathways or on cell cycles, mainly hampering S phase or G1 phase (Fig. S6).

**Figure 5:**
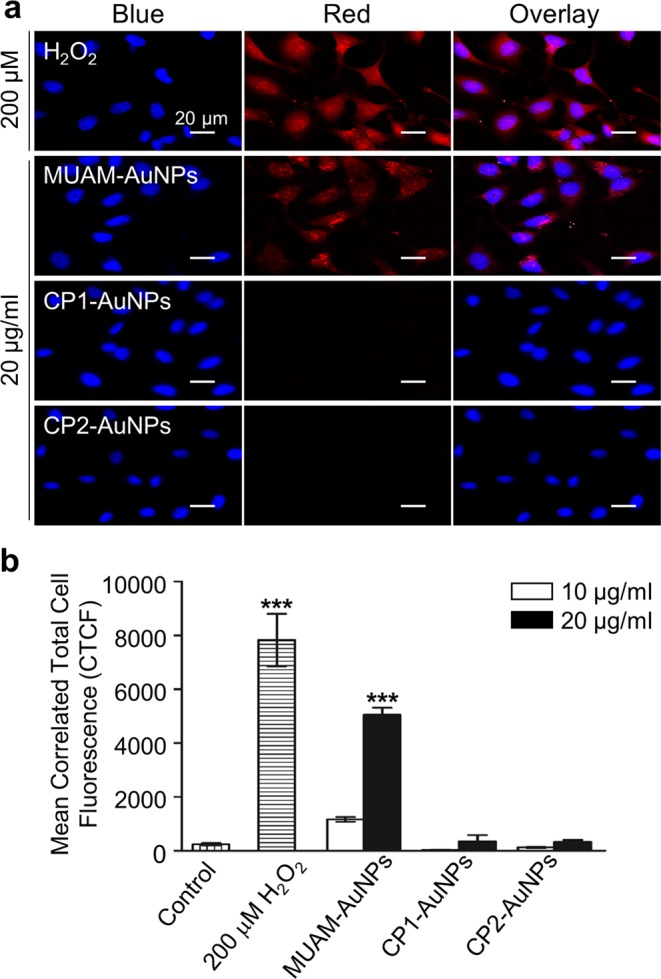
Reactive oxygen species generation induced by cationic gold nanoparticles (AuNPs). (**a**) The level of ROS generation in cationic AuNPs-treated cells were monitored. MUAM-AuNPs treated cells showed increased ROS level comparable to H_2_O_2_-treated control group, while other cationic AuNPs did not induce noticeable ROS generation. Scale bar: 20 μm. (**b**) The fluorescence intensity shows the amount of ROS in AuNPs-treated cells. The level of ROS increased as the concentration of MUAM-AuNPs increased (***p < *0.001*, one-way ANOVA).

We then examined the damage on genomic DNA mediated by modified AuNPs using the comet assay. The genomic DNAs were isolated from AuNPs-treated cells and analysed using electrophoresis. Tail moment and % tail DNA measurement indicated significant DNA damage in MUAM-AuNPs treated samples (Figs 4c,d and S7). Other AuNPs did not apply noticeable damages. As the oxidative stress could cause the DNA damage^53^, we examined the levels of ROS induced by various cationic AuNPs. ROS generation in AuNPs-treated cells were monitored and only MUAM-AuNPs treated cells showed increased ROS level which is comparable to H_2_O_2_-treated control group (Fig. 5). The level of ROS increased with the concentration of MUAM-AuNPs. Interestingly, MUAM-AuNPs increased ROS in perinuclear region, while the ROS induced by H_2_O_2_ were observed throughout the cytoplasm. The fluorescence microscopy showed that perinuclear localization of internalized MUAM-AuNPs (Fig. 3c) suggesting that MUAM-AuNPs probably induced DNA damage via oxidative stress. We next tested if we can moderate the cytotoxicity induced by MUAM-AuNPs by treating cells with a reducing agent, glutathione (GSH). The GSH was adopted in our experiment because it is the major chemical participating in the cellular redox reaction^54^. The results showed that pre-treatment with GSH delayed cell death of MUAM-AuNPs treated cells (Fig. S8).

These studies together demonstrate that MUAM-AuNPs inhibited the cell proliferation at LD_0_ by altering multiple cellular functions and behaviours. MUAM-AuNPs affected cells at least by three different routes. First, they altered the cytoskeletal structure by directly interfering with actin polymerization reaction, resulting in delayed cell motility. Second, they caused severe damage on genomic DNA by generating ROS near nucleus. Third, they affected the DNA replication pathways indirectly, resulting in inhibited DNA replication.

### What is structural characteristics of toxic AuNPs?

We have assessed the effects of modified AuNPs on cells and learned that the MUAM-AuNPs showed unique toxicity. Seemingly, the results are compatible to the previous reports suggesting that some cationic AuNPs are cytotoxic. Thus it is still necessary to explain why only MUAM-AuNPs shows strong cytotoxicity among several cationic AuNPs. We investigated the effect of different surface chemistry of MUAM-AuNPs; i.e., the primary amine functional group, the magnitude of positive charge, and the hydrophobic moiety neighbouring the positively charged functional group.

The first consideration for the structural basis of toxicity was the primary amine functional group. The association of the primary amine groups with toxicity has been reported previously^55,56^. Our results, however, suggest otherwise. Among two modified-AuNPs carrying primary amine groups, namely MUAM-AuNPs and CP2-AuNPs, only MUAM-AuNPs showed substantial cytotoxicity.

We then speculated that the distinguishing cytotoxicity of MUAM-AuNPs could be due to the larger magnitude of positive charge. The interaction between cationic AuNPs and negatively charged plasma membrane has been reported as the origin of toxicity by several groups^10,22^. MUAM-AuNPs has the most positive ζ-potential of 41.8 ± 3.8 mV, while the ζ-potentials of CP1-AuNPs and CP2-AuNPs are 31.4 ± 1.5 mV and 27.8 ± 3.8 mV, respectively. We observed that all three cationic AuNPs are located near the plasma membrane while neutral or anionic AuNPs were rarely found in or near the mammalian cells (Fig. 3c, black arrows). Studies using field emission scanning electron microscopy (FE-SEM) and confocal microscopy provided consistent results showing that all three cationic AuNPs were internalized with MUAM-AuNPs being more effective (Figs 3c and S9). By looking at the interaction with plasma membrane and consequent internalization, the magnitude of positive charge appears to be an important factor determining the toxicity. In order to test this hypothesis, we prepared MUAM-AuNPs with a lower density of amines on the surface, namely MUAM1-AuNPs and MUAM2-AuNPs. MUAM1-AuNPs and MUAM2-AuNPs reported ζ-potential values of 33.9 ± 3.5 mV and 29.7 ± 0.8 mV, respectively, which are comparable to CP1-AuNPs and CP2-AuNPs. Cells treated with MUAM1-AuNPs and MUAM2-AuNPs were subjected to MTT viability assay, individually. To our surprise, MUAM1-AuNPs and MUAM2-AuNPs gave LD_50_ values similar to MUAM-AuNPs (Fig. 2d). This result strongly suggests that the magnitude of positive charge is not the critical factor that determines the cytotoxicity.

We then paid attention to the hydrophobic moieties in cationic AuNPs. Earlier, Rotello and his group suggested that increased hydrophobic moiety is associated with the increase in cellular uptake as well as cytotoxicity in serum free media^57^. As MUAM carries both cationic head groups and hydrophobic moieties, we were curious whether this structural characteristics of MUAM could be the basis of cytotoxicity. We considered two above-mentioned data, AuNP internalization assay and trypan blue assay. These data showed that all three cationic AuNPs internalize but only MUAM-AuNPs compromise membrane integrity, suggesting that the association between MUAM-AuNPs and the plasma membrane is lytic, while CP1-AuNPs and CP2-AuNPs are more penetrating. In addition, the lysosomal membrane integrity assay using Lucifer yellow^58^ also showed that MUAM-AuNPs induced impairment of lysosome membranes (Fig. S10). In order to verify this idea, we prepared two modified CP1 peptides, CP1M1 and CP1M2, by introducing hydrophobic moieties to hydrophilic CP1 peptide (Fig. S11). FE-SEM data showed that the hydrophobic moiety increased the cellular uptake of cationic AuNPs in MUAM1-, MUAM2-, CP1M1-, and CP1M2-AuNPs to the level of MUAM-AuNPs (Fig. S9). The MTT viability assay with CP1M1- and CP1M2-AuNPs treated cells demonstrated increased cell death with the introduction of a hydrophobic moiety (Fig. 2e) and the generation of ROS was also monitored in CP1M1- and CP1M2-AuNPs treated cells (Fig. S12). We reasoned that hydrophobic chain of cationic AuNPs could help cellular internalization through the interaction with the surrounding lipid molecules in the plasma membrane^59^. In the process, pores can be created in the cell membranes leading to cellular toxicity by destroying the delicate concentration balance of intracellular versus extracellular ions, proteins, and other important macromolecules that are required to protect the cell integrity and functions.

Additionally, we considered the effect of protein corona at the interface of AuNPs and biological components, as a factor influencing the biological effect of AuNPs. The formation of protein corona was monitored using DLS, ζ-potential measurement, and gel-electrophoresis for cationic AuNPs (Fig. S13). The rapid formation of soft corona and following protein exchange to form hard corona was observed from MUAM-AuNPs, while the CP1- and CP2-AuNPs showed slower hard corona formation. The size of AuNPs converged to 100–200 nm in 48 h with small PDI values suggesting that the AuNPs maintained well-dispersed states in culture media. These results are compatible with previous reports suggesting that the hydrophobic ligands increases absorption kinetics and absorbed protein quantity compared with hydrophilic ligands^60–62^. Considering the time scale, MUAM-AuNPs seemed to interact with cells when it is coated with soft corona and their surface can be exposed to interact with the biological targets, i.e. plasma membranes^63^.

### Down regulation of Cell cycle-related genes in MUAM-AuNPs treated cells

**Figure 6:**
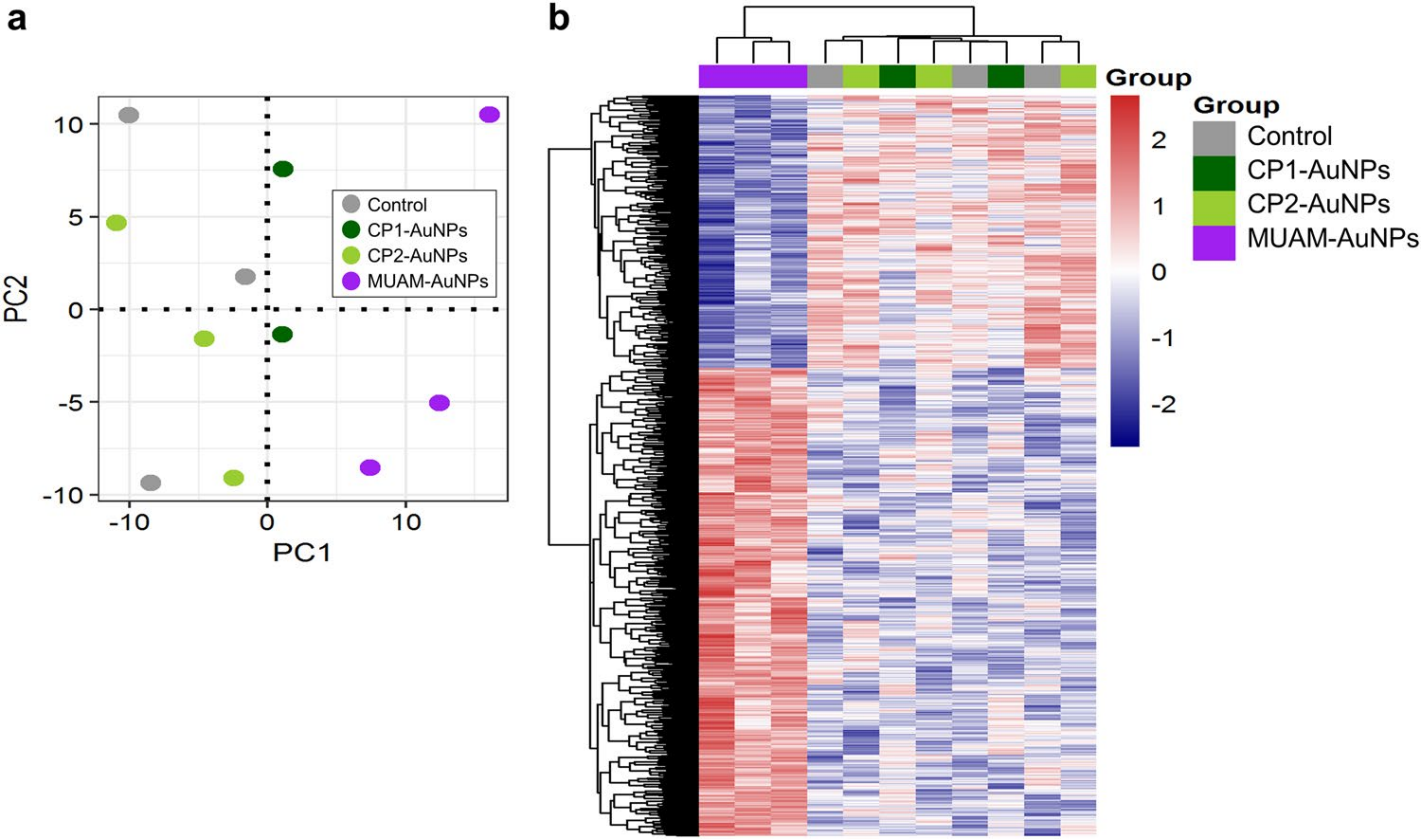
Gene expression profiles of HeLa cells treated with cationic gold nanoparticles (AuNPs) (**a**) Global gene expression profiles of the cationic AuNPs (MUAM-, CP1-, and CP2-AuNPs) treated cells were analysed by principal component analysis. The MUAM-AuNPs treated cells showed unique gene expression patterns, while the CP1- and CP2-AuNPs treated samples showed the patterns similar to the control. (**b**) Heat map analysis of gene expression pattern shows hierarchical clustering of 1,156 differentially expressed genes between MUAM-AuNPs treated samples and the group of three other samples. Red and blue colours indicate up- and down-expression levels, respectively.

To understand the underlying mechanisms of cytotoxicity, gene expression analysis was performed. The mRNA level of cells treated with CP1-, CP2-, and MUAM-AuNPs were analysed using the human genome microarray. Untreated cells were also analysed as a control. The principal component analysis (PCA) showed that the MUAM-AuNPs altered the global gene expression pattern in a unique way compared with non-treated control or other cationic AuNPs-treated samples (Fig. 6a). The following differentially expressed genes (DEGs) analysis on MUAM-AuNPs treated samples in comparison to the set of three other samples revealed that 1,156 differentially expressed genes were identified, including 730 up-regulated and 426 down-regulated genes, which were involved in cellular metabolic process, protein catabolic process, cell cycle (*p* < *0.001*), and G1/S phase transition (*p* < *0.05*) (Fig. S14). Heatmap visualization of DEGs also showed a unique pattern of MUAM-AuNPs treated samples compared with the rest (Fig. 6b). It is notable that the cell cycle related genes, especially the genes involved in G1 phase, as well as genes involved in nucleic metabolic process are down regulated. During G1 phase, growth activity promotes DNA replication and initiates G1-to-S phase transition^64^. Thus the down-regulation of G1 phase genes is well correlated with aforementioned inhibition of DNA synthesis in MUAM-AuNPs treated cells. The result suggests that the inhibited cell proliferation of MUAM-AuNPs treated cells in CFE assay is probably due to the repressed transition from G1-to-S phase in cell cycle. The protein-protein interaction (PPI) networks were constructed to understand the biological interactions among the DEGs (Fig. S15a). The cell cycle related proteins TAF1 and PTEN were hub proteins that have high neighborhood connectivity, where TAF1 is TATA-box binding protein associated factor 1 and has 27 degree and PTEN is phosphatase and tensin homolog with 24 degree. A sub-network was identified using these proteins (Fig. S15b).

## Conclusion

We have studied the structural origin of AuNPs-induced cytotoxicity using well-tailored charge library of AuNPs. We observed that the cationic AuNPs exhibit a range of cytotoxicity stretching from being nontoxic to being severely toxic while neutral and anionic AuNPs were not noticeably cytotoxic. As MUAM-AuNPs exhibited unique toxicity, we determined the characteristics of the toxic AuNPs by comparing with multiple AuNPs, in which their structural properties, such as the magnitude (density) of charge, surface functional groups, and hydrophobicity, are modulated. From these studies, we concluded that the positive charge neighbouring the hydrophobic moiety is the structural characteristics of the toxic AuNPs while the magnitude of charge or the amine functional group were not the sole factor gave rise to the cytotoxicity.

We also tried to understand the mechanistic aspect of MUAM-AuNPs induced cytotoxicity on mammalian cells. MUAM-AuNPs showed acute toxicity with LD50 values of approximately 16 μg/ml and affected various cellular functions at LD0 potentially evoking the secondary or long-term damage on cells or tissues. MUAM-AuNPs affected the cell functions by the following routes: (i) distorted cytoskeletal structures via direct interaction with F-actins; (ii) inhibited DNA replication by down-regulating the related gene expression; (iii) caused DNA damage through ROS generation.

Through this work, we identified the structural characteristics responsible for the AuNPs-induced cytotoxicity and provided the mechanistic explanations by implementing a systematic toxicological evaluation using charge library of modified AuNPs. There are still needs for continual research to identify other physicochemical properties that controls the biological effect of nanoparticles, to explore the structure-toxicity relationship of nanoparticles, and to exploit these findings for manufacturing of safe nanoparticles for clinical use.

## Methods

### Chemicals and materials

Protected amino acids and resins were purchased from NovaBiochem (Darmstadt, Germany) and AnaSpec (Fremont, CA). Mesna, MUAM, HAuCl_4_∙3H_2_O, and sodium citrates were products of Sigma Aldrich (St. Louis, MO). mPEG_350_-SH was purchased from Nanocs (Boston, MA). Dulbecco’s Modified Eagle’s Medium (DMEM), penicillin-streptomycin, Trypsin-EDTA, and fetal bovine serum (FBS) were purchased from Welgene (Daegu, Korea). Trypan blue stain was from Life Technologies (Carlsbad, CA). Cell Proliferation Kit I (MTT) was purchased from Roche (Mannheim, Germany). Actin Polymerization Biochem Kit, CometAssay® Kit, Actin-Toolkit, BrdU Cell Proliferation Assay Kit, and Cellular Reactive Oxygen Species Detection Assay Kit were purchased from Cytoskeleton Inc. (Denver, CO), Trevigen (Gaithersburg, MD), Hypermol (Bielefeld, Germany), Calbiochem® (Darmstadt, Germany), and abcam (Cambridge, MA), respectively. Ultrapure water (18.3 MΩ•cm) was generated using the Pure power I water system from Daihan Scientific (Wonju, Korea) and used for all the experiments. Synthetic peptide ligands were analysed using a 2796 Alliance high-performance liquid chromatography (HPLC) BioSystem equipped with a 2487 dual λ absorbance detector, and ZQ single quadrupole mass spectrometer from Waters (Milford, MA). All analysis were performed on Vydac C18 columns (5 micron, 4.6 × 150 mm) from Grace (Columbia, MD) using linear gradients of solvent A (0.1% aqueous formic acid) vs. solvent B (90% acetonitrile, 10% double distilled water (ddH_2_O), 0.1% formic acid) at a flow rate of 0.3 ml/min. ζ-potential and the size distribution of the modified AuNPs were measured using Zetasizer Nano ZS90 from Malvern (Worcestershire, UK). UV-Vis spectra of modified AuNPs was measured by using a NanoDrop 2000c from Thermo Fisher (Waltham, MA). Fluorescence images were acquired by an Eclipse Ti confocal laser scanning microscope from Nikon Instruments (Tokyo, Japan). The morphology of AuNPs and cellular uptake of AuNPs were observed using Sigma FE-SEM from Zeiss (Jena, Germany).

### Preparation of AuNPs

AuNPs of 20 nm were prepared following a standard protocol introduced by Turkevich^65^. Briefly, an aqueous solution of 0.3 mM HAuCl_4_∙3H_2_O (50 ml) was brought to boil with vigorous stirring. To this solution was added 5 ml of 10 mM sodium citrate solution. The mixture turned blue within 25 s and then changed to red-violet in 70 s. After an additional boiling for 10 min, the heating source was removed and the colloid was stirred for another 15 min. The resulting solution of AuNPs was characterized by UV-Vis spectroscopy and an absorbance maximum at 520 nm was obtained. DLS indicated that the average particle size was 22.1 ± 0.5 nm at 25 °C and the average surface charge was −54.2 ± 3.1 mV. The size and morphology of AuNPs were analysed by using FE-SEM operating at 5 to 15 kV.

### Functionalization and characterization of AuNPs

Citrate-coated AuNPs were functionalized using thiol ligands by modified place-exchange reaction^66^. Briefly, an aqueous solution of primary mPEG-ligand (10 mM, 100 μl) was added to the solution of citrate-coated AuNPs (28.3 μg/ml, 2 ml) and incubated for 24 h at 4 °C. The supernatant was removed by centrifugation and the secondary ligands (500 μM, 1 ml) was added to the mPEG-coated AuNPs. After 24 h treatment, the supernatant was removed by centrifugation and the modified AuNPs were washed and resuspended in ddH_2_O. Modified AuNPs were characterized by using UV-Vis spectroscopy, DLS, and ζ-potential measurement (at pH 7). All experiments were performed at least three times. The results were presented as the mean ± standard deviation (SD). For the rest of the paper, modified AuNPs are referred to as secondary ligand name-AuNPs.

### Viability assay

Viability assays were performed using two human cell lines, HeLa and NHDF. Cells were treated with modified AuNPs for 24 h and were subjected to both colorimetric MTT and trypan blue assays. To determine the relative cell viability by MTT assay, 1 × 10^4^ cells were plated per well in a 96-well plate and cultured for 24 h in DMEM containing 10% FBS (complete culture media, CCM). The culture medium was replaced with DMEM containing 1% FBS (D1 medium) and the cells were treated with modified AuNPs at a series of concentrations; 0, 0.028, 0.28, 2.8, 28, and 100 μg/ml. After 24 h exposure, the viability was determined using Cell proliferation kit I according to the vendor’s protocol. Data were reported as the means of three independent experiments (three replicates each) ± standard error of mean^52^ and were expressed as percent viability with respect to the solvent control.

For the trypan blue assay, 4 × 10^4^ cells were seeded in each well of 6-well plate and cultured for 24 h in CCM. The culture medium was replaced with D1 medium and the cells were exposed to 0, 0.028, 0.28, 2.8, 28, and 100 μg/ml of modified-AuNPs, individually. After 24 h exposure, the cells were washed with PBS, detached with 0.5 ml of trypsin-EDTA solution, and were harvested using 1 ml of CCM. Each sample was treated with trypan blue and the stained cells were counted using a hemocytometer. Data were reported as the means of three independent experiments (three replicates each) ± SEM and expressed as percent viability with respect to the solvent control.

### Cell motility assay

A capillary (1.1 mm diameter) was fixed onto each well of a 12-well culture plate using sterile vacuum grease. HeLa cells (8 × 10^4^) were plated in each well and incubated for 12 h to adhere to the surface in CCM. The medium was replaced with 1 ml of D1 medium and modified AuNPs were added to make a final concentration of 10 μg/ml. After 24 h exposure, the medium was replaced with fresh CCM and the capillary was carefully removed to reveal a gap. Images were taken at 0 and 24 h using a microscope and the number of cells migrated into the gap was counted using NIH ImageJ analysis software (ver. 1.48). The results were reported as percent migration with respect to the solvent control.

### Visualization of cytoskeletal structures and internalized AuNPs

HeLa cells were grown on a cover glass till they reached 80% confluence in CCM. The cells were washed and treated with AuNPs (10 μg/ml) for 24 h in D1 medium. The filamentous actins were visualized by staining with Alexa Fluor 568 phalloidin from Invitrogen (Carlsbad, CA) according to the manufacturer’s protocol. The nucleus was stained with DAPI. The stained cells were imaged using the confocal fluorescence microscopy. Fluorescent images were obtained to study the cytoskeletal structure and the phase contrast images were obtained to monitor the cellular uptake of AuNPs.

### Effect of AuNPs on *in vitro* actin polymerization

The *in vitro* actin polymerization assay was carried out with Actin Polymerization Biochem Kit according to the manufacturer’s protocols. The solution of G-actin monomer (0.08 mg/ml) was incubated on ice for 1 h and centrifuged for 30 min, 14,000 rpm at 4 °C to depolymerize any remaining oligomers in solution. Modified AuNPs were added to 180 μl G-actin solution to make final concentration of 10 μg/ml. Actin polymerization buffer (500 mM KCl, 20 mM MgCl2, 0.05 M guanidine carbonate, and 10 mM ATP, 20 μl) was added to each well and the fluorescence signal was observed every 30 s for 1 h, using Tecan F200pro microplate reader (Männedorf, Switzerland). The experiment was carried out three times in triplicates.

### CFE assay

HeLa cells were seeded at a density of 200 cells per dish (60 mm in diameter) and cultured in CCM for 24 h (day 1). The CCM was replaced with 2 ml of D1 medium and modified AuNPs were added to make a final concentration of 10 μg/ml. After 24 h of exposure, the D1 medium was replaced with fresh CCM. At day 8, cells were fixed with 3.7% (v/v) formaldehyde solution in PBS and stained with 10% (v/v) Giemsa stain solution. Colonies of over 50 cells were counted under a microscope and the number of colonies were reported as means of three independent experiments (three replicates each) ± SEM and were expressed as percent CFE with respect to the solvent control.

### DNA replication assay

The DNA replication assay was carried out using BrdU Cell Proliferation Assay Kit according to the manufacturer’s instruction. HeLa cells (1 × 10^4^ cells/well) were treated with modified AuNPs at a final concentration of 10 μg/ml in D1 medium for 24 h, then treated with BrdU for another 24 h. The cells were fixed and treated with anti-BrdU antibody-HRP conjugate. Then the HRP activity was monitored by measuring the absorbance at 450 nm. The average of three independent experiments in triplicate were reported.

### ROS detection and quantitation

The assay was performed on cells treated with modified AuNPs (20 μg/ml) for 24 h. A batch of cells treated with H_2_O_2_ (200 μM) and a batch of non-treated cells were included as positive and negative controls, respectively. AuNPs-treated cells and controls were incubated in ROS Deep Red working solution for 1 h. Fluorescence from oxidized reagents was imaged using the fluorescence microscope. Fluorescent images were analysed with ImageJ and processed as previously reported^67^. Briefly, the total fluorescence intensity of each cell (Integrated density, IntDen) was determined using ImageJ. The background was obtained by measuring the fluorescence intensity of regions out of the cells. The corrected total cell fluorescence (CTCF) was then determined by subtracting the background from the integrated density, by using the following equation:

$${\rm{CTCF}}=\,{\rm{Int}}\,{\rm{Den}}\,-\,({\rm{Area}}\,{\rm{of}}\,{\rm{selected}}\,{\rm{cell}}\times {\rm{Mean}}\,{\rm{fluorescence}}\,{\rm{of}}\,{\rm{background}})$$

### Comet assay

DNA damage in HeLa cells was examined using CometAssay® Kit according to the manufacturer’s instructions. Cells were plated (1 × 10^5^ cells/well) in 12-well culture plates and incubated for 12 h in CCM. The medium was replaced with 1 ml of D1 medium and modified AuNPs were added to make a final concentration of 10 μg/ml. After 24 h exposure, cells were washed with PBS twice and scraped using a cell scraper. Cells were collected and embedded in Comet LMAgarose (1% low-temperature melting agarose). The agarose gel was transferred to CometSlide™ and kept at 4 °C for 10 min. Slides were placed in a cold lysis solution and kept in the dark overnight. Electrophoresis was performed in alkaline electrophoresis solution (200 mM NaOH, and 1 mM EDTA at pH 13.0) for 30 min at 20 V, 300 mA. The slides were then neutralized and stained with SYBR Green dye. At least 50 comets per slide were imaged under fluorescence microscopy to measure the tail moment and % tail DNA.

### RNA extraction and microarray assay

HeLa cells were seeded in 35 mm culture dishes at density of 4 × 10^5^ cells per dish and incubated in CCM for 24 h. The culture medium was replaced with D1 medium and the cells were exposed to modified AuNPs (10 μg/ml for MUAM-AuNPs, 50 μg/ml for CP1- and CP2-AuNPs). Solvent-treated cells were included as a control. After 12 h exposure, the medium was removed and the total RNA was extracted from cells. Total RNA was then submitted to the Macrogen Inc. (Seoul, Korea) where RNA quality was analysed and microarray assay was performed using Human Gene 2.0 ST Array from Affymetrix (Santa Clara, CA).

### Gene expression profiling, Gene Ontology (GO) enrichment, and network analysis

The microarray datasets were summarized and normalized with robust multi-average (RMA) method using oligo R package^68^. The dataset was adjusted for batch effects using ComBat^69^ function in surrogate variable analysis (SVA) R package^70^ and PCA was performed. The dataset was then analysed to identify the DEGs between MUAM-AuNPs treated samples and other three samples (negative control, CP1-AuNPs treated and CP2-AuNPs treated samples) using limma R package^71^. Functional enrichment analysis based on GO category were performed on up- and down-regulated DEGs, using the Database for Annotation, Visualization, and Integrated Discovery (DAVID)^72^. The PPI network analysis was performed using the STRING database (v10.5)^73^.

## Supplementary information

Supplementary information
