# Supplementary material for: Molecular origin of AuNPs-induced cytotoxicity and mechanistic study
Source: Sci Rep. 2019 Feb 21;9:2494. doi: 10.1038/s41598-019-39579-3 (PMC6385177; doi:10.1038/s41598-019-39579-3)
Supplement: Supplementary file 1 — Supplementary information [file 41598_2019_39579_MOESM1_ESM.docx]

**Supplementary information**

**Molecular origin of AuNPs-induced cytotoxicity and mechanistic study**

Euiyeon Lee^1^, Hyunjin Jeon^1^, Minhyung Lee^1^, Jeahee Ryu^1^, Chungwon Kang^1^, Soyoun Kim^1^, Junghyun Jung^2^ and Youngeun Kwon^1^*

*^1^Department of Biomedical Engineering (BK21 plus); ^2^Department of Life Science, Dongguk University, Pildong 3-ga, Seoul 04620, Korea*

**Address for correspondence:*

*Youngeun Kwon, Department of Biomedical Engineering, Dongguk University, Seoul, Korea. Tel: +82-31-961-5151. E-mail:* [*ykwon@dongguk.edu*](mailto:ykwon@dongguk.edu)

**Table of contents**

**Supplementary methods** **Page #**

Synthesis of peptide ligands and characterization 3

Effect of AuNPs on *in vitro* actin polymerization (Fluorescence microscope) 4

Monitoring the cellular uptake of AuNPs using FE-SEM 4

Treatment of reducing agents to moderate AuNPs-induced cytotoxicity 4

**Supplementary Figure**

Supplementary Figure S1 6

Supplementary Figure S2 6

Supplementary Figure S3 7

Supplementary Figure S4 8

Supplementary Figure S5 8

Supplementary Figure S6 9

Supplementary Figure S7 9

Supplementary Figure S8 10

Supplementary Figure S9 10

Supplementary Figure S10 11

Supplementary Figure S11 11

Supplementary Figure S12 12

Supplementary Figure S13 12

Supplementary Figure S14 13

Supplementary Figure S15 14

**Supplementary methods**

**Synthesis of peptide ligands and characterization**

The peptides shown in Table 1 were synthesized at a scale of 0.1 mmol on a rink amide AM resin according to the standard Fmoc-based solid phase peptide synthesis strategy (Raymond B et al., 2012). Briefly, the Fmoc protecting group was removed from the resin by treating the resin with 20% piperidine in DMF (v/v) for 15 min, three times. Fmoc-amino acids (0.2 mmol) were activated by adding HBTU (0.18 mmol) and DIPEA (0.4 mmol) in DMF and the mixture was added to the resin. Coupling reaction was carried out for 1 h. Following chain assembly, global deprotection of peptide and cleavage from the support were accomplished by treating with a cleavage cocktail (90% TFA, 5% Ethanethiol, 2.5% H_2_O, and 2.5% Triisopropylsilane) for 3 h at room temperature (RT). The crude peptide products were precipitated and washed with anhydrous cold diethylether. The crude peptides were purified using Waters Sep Pak C18 Cartridges and identified by ESI-MS to obtain the following twelve peptides;

Anionic peptide 1 (**AP1**, Ac-DDDYC). The purified peptide was characterized as the desired product by LC/MS [Expected mass=670.2 Da; observed mass= 669.3 Da]

Anionic peptide 2 (**AP2**, Ac-EEEGYC). The purified peptide was characterized as the desired product by LC/MS [Expected mass=769.3 Da; observed mass=770.8 Da]

Anionic peptide 3 (**AP3**, Ac-DDDGYC). The purified peptide was characterized as the desired product by LC/MS [Expected mass=727.2 Da; observed mass= 729.0 Da]

Neutral peptide 1 (**NP1**, DDDYC). The purified peptide was characterized as the desired product by LC/MS [Expected mass=628.2 Da; observed mass= 627.2 Da]

Neutral peptide 2 (**NP2**, AAAGYC). The purified peptide was characterized as the desired product by LC/MS [Expected mass=553.2 Da; observed mass= 553.8 Da]

Neutral peptide 3 (**NP3**, Ac-SSSGYC). The purified peptide was characterized as the desired product by LC/MS [Expected mass=643.2 Da; observed mass=644.9 Da]

Cationic peptide 1 (**CP1**, RRRGYC). The purified peptide was characterized as the desired product by LC/MS [Expected mass= 808.4 Da; observed mass= 807.4 Da]

Cationic peptide 2 (**CP2**, KKKGYC). The purified peptide was characterized as the desired product by LC/MS [Expected mass=724.4 Da; observed mass= 725.4 Da]

Cationic peptide 3 (**CP3**, RGYC). The purified peptide was characterized as the desired product by LC/MS [Expected mass= 496.2 Da; observed mass= 495.2 Da]

Cationic peptide 4 (**CP4**, KGYC). The purified peptide was characterized as the desired product by LC/MS [Expected mass=468.2 Da; observed mass= 469.3 Da]

Modified CP1-1 (**CP1M1**, RRRGY-Ahx-C). The purified peptide was characterized as the desired product by LC/MS [Expected mass= 921.5 Da; observed mass=922.9 Da]

Modified CP1-2 (**CP1M2**, RRRGYKC_11_C). The purified peptide was characterized as the desired product by LC/MS [Expected mass=1104.7 Da; observed mass=1105.8 Da]

**Effect of AuNPs on *in vitro* actin polymerization (Fluorescence microscopy)**

The *in vitro* actin polymerization assay was carried out with Actin-Toolkit according to the manufacturer’s protocols. Modified AuNPs were added to Atto488-G-actin solution (0.05 mg/ml, 50 μl) to make final concentration of 10 μg/ml. PolyMix buffer (1 M KCl, 20 mM MgCl_2_, 0.1 M imidazole, and 10 mM ATP at pH 7.4, 50 μl) was added to each sample and the fluorescent signal was observed for 30 min using the fluorescence microscope.

**Monitoring the cellular uptake of AuNPs using FE-SEM**

HeLa cells treated with AuNPs were grown on cover glasses, fixed with 3% glutaraldehyde in PBS, and then washed three times with PBS. The samples were dehydrated using aqueous methanol (30-100%, v/v) and then were dried overnight at RT. The samples were platinum-coated to avoid charging effect. Images were acquired at voltages ranging from 5 to 15 kV using SIGMA FE-SEM.

**Treatment of reducing agents to moderate AuNPs-induced cytotoxicity**

HeLa cells (1×10^4^ cells/well) were plated in 96-well plates in CCM and were incubated for 24 h. The medium was replaced with fresh CCM containing 1 mM glutathione (GSH) and cells were incubated for 3 h. The culture medium was replaced with D1 medium containing 1 mM GSH and the cells were treated with MUAM-AuNPs to make a final concentration of 28 μg/ml. The MUAM-AuNPs-treated cells without GSH treatment and solvent-treated cells were included as positive and negative controls, respectively. The viability of cells were determined at varying time points using MTT assay. Data were reported as the means of three independent experiments (three replicates each) ± standard error of mean (SEM) and were expressed as percent viability with respect to the solvent control.

**Formation of protein corona on modified AuNPs**

CCM consisted of DMEM supplemented with 1, and 10% of FBS. Modified AuNPs and CCM were mixed (1:10 v/v) and placed in an incubator at room temperature for different incubation times. As a result, the final concentration of AuNPs was 57 μg/ml, which is the absorbance at 1. The CCMs used were DMEM high glucose with 4500 mg/L D-glucose, 110 mg/L sodium pyruvate, sodium bicarbonate, L-glutamine, and phenol red, and sterile-filtered after supplementing with FBS, penicillin/streptomycin. Cationic AuNPs formed with protein corona are measured through DLS and zeta potential.

**References**

Raymond B, Peter W, John O. 2016. Advances in Fmoc solid-phase peptide synthesis. J Pept Sci 22:4-27.

**Supplementary figures**


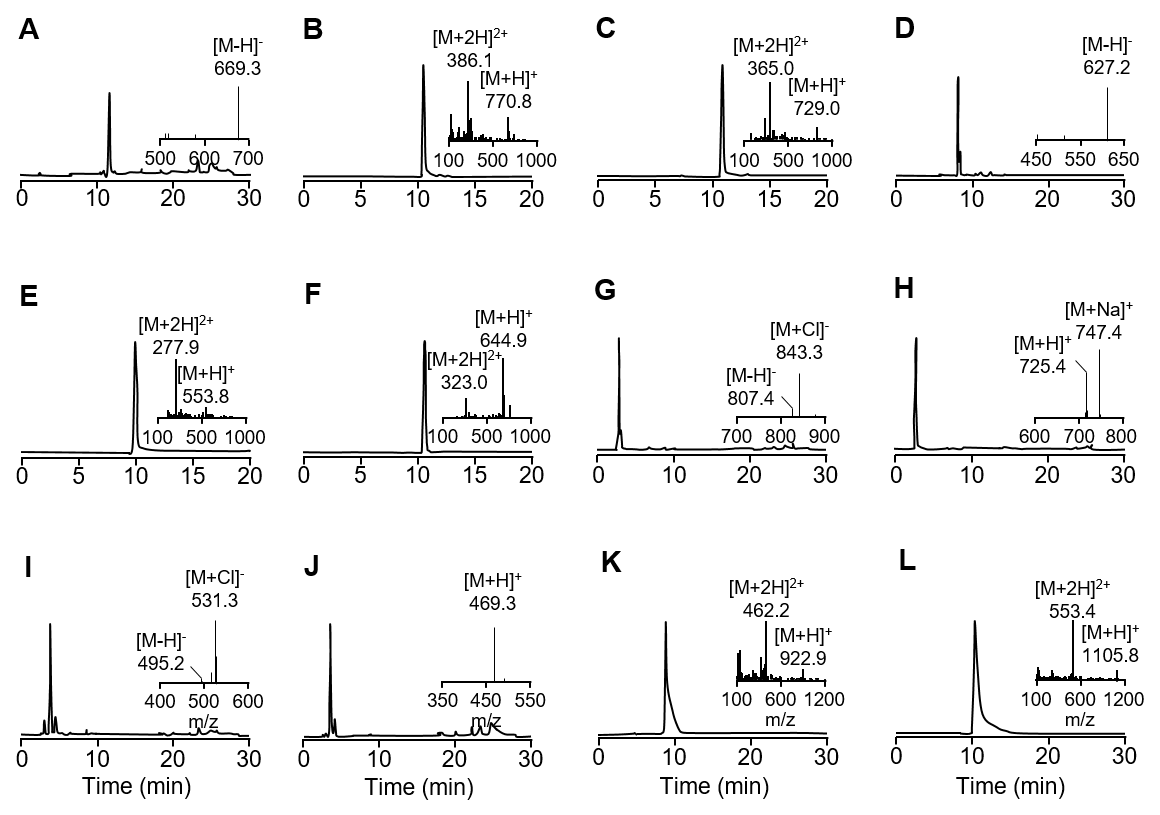

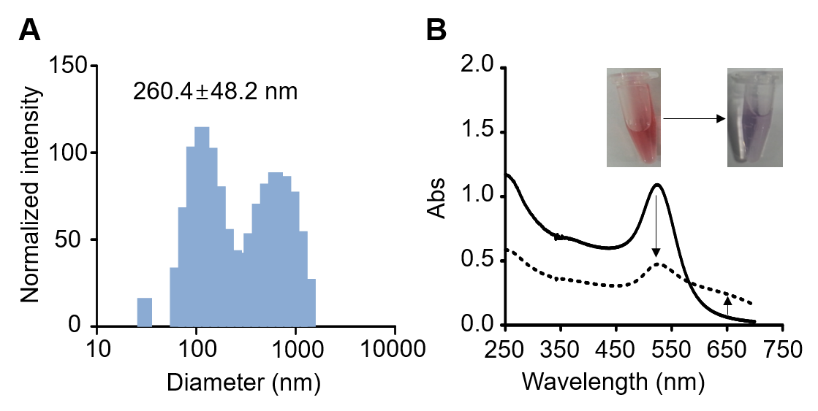


Supplementary Figure S1. Analytical High performance liquid chromatography (HPLC) and mass spectrometry data for synthetic peptide ligands. HPLC chromatograms and ion masses (insets) were obtained for synthetic peptides AP1 (A), AP2 (B), AP3 (C), NP1 (D), NP2 (E), NP3 (F), CP1 (G), CP2 (H), CP3 (I), CP4 (J), CP1M1 (K) and CP1M2 (L). All peptide ligands were purified by HPLC using a linear gradient of 0-50 % of ACN over 20-30 min.

Supplementary Figure S2. Aggregation of cationic gold nanoparticles (AuNPs). The addition of MUAM ligands to citrate-capped AuNPs caused instant aggregation of AuNPs. (A) Dynamic laser scattering showed a significant increase in size. (B) UV-Vis spectra of AuNPs in citrate (solid line) and in MUAM (dashed line) are compared. Insets are the photography of AuNPs in citrate and MUAM (right; blue color indicates aggregation of AuNPs).


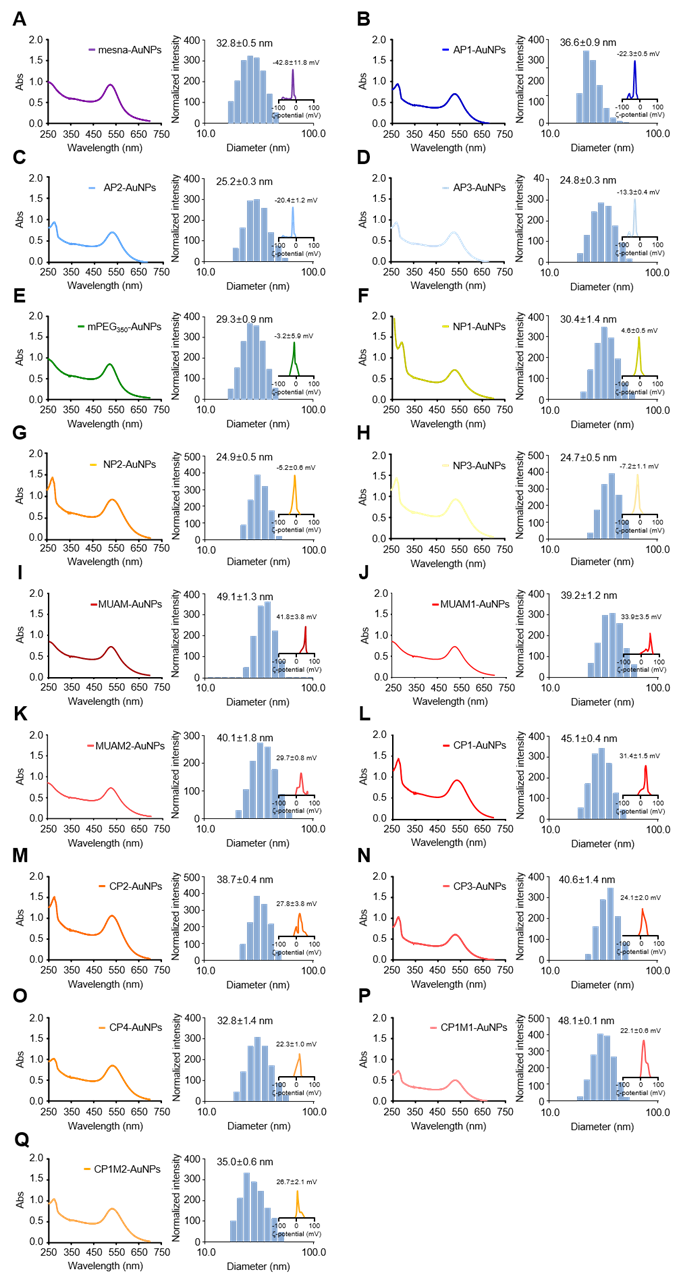


Supplementary Figure S3. Characterization of modified gold nanoparticles (AuNPs). The modified AuNPs were modified and analysed using UV-Vis spectroscopy, dynamic laser scattering and zeta potential (inset) measurements.


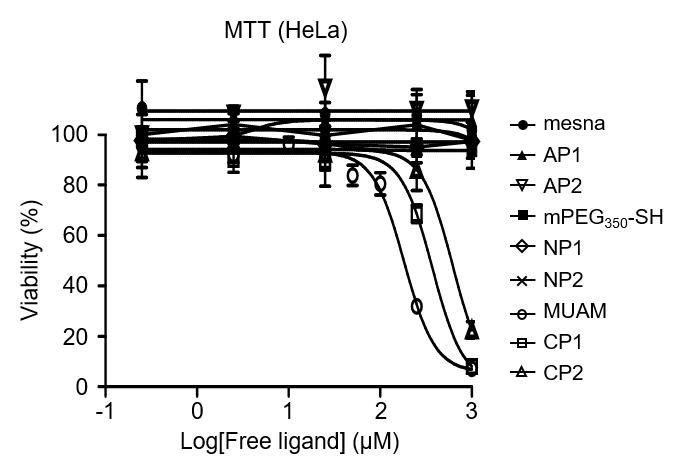


**Supplementary Figure S4.** Effect of free ligands on cell viability. MTT assay was performed on HeLa cells treated the various free ligand for 24 h.


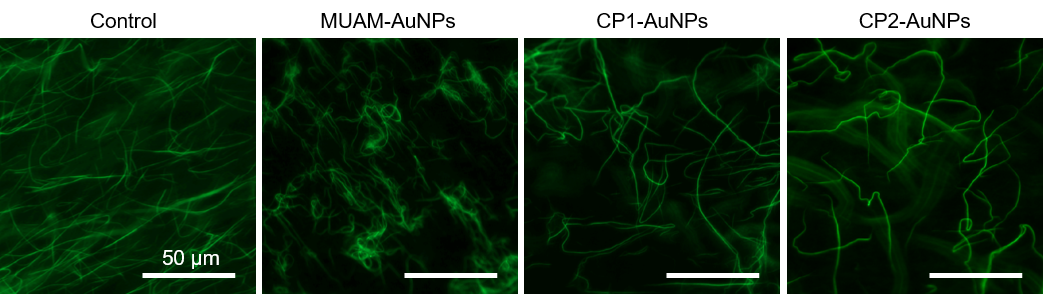


Supplementary Figure S5. *In vitro* polymerization of actin (0.05 mg/ml, Atto488-labelled) in the presence of modified AuNPs at LD_0_ (10 μg/ml). Images was captured 30 min after initiation of actin polymerization.


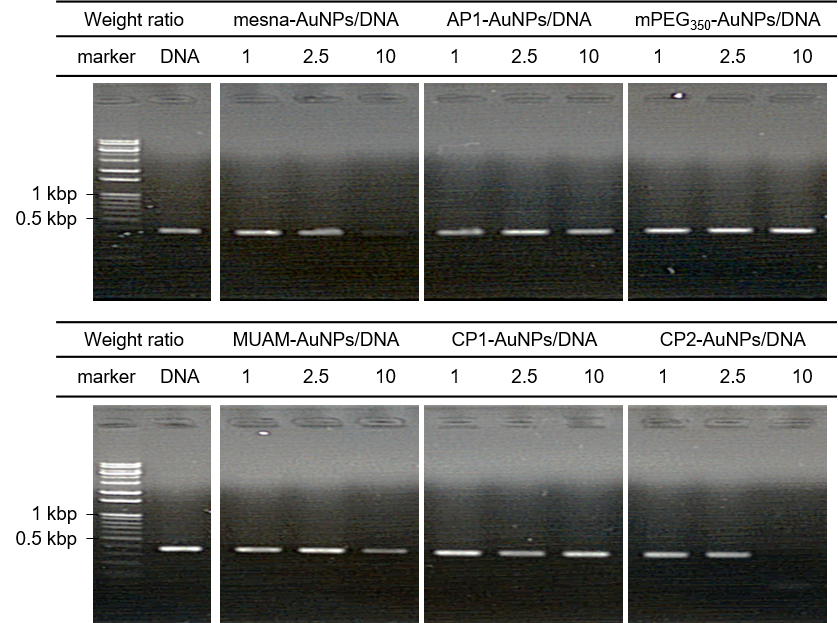


Supplementary Figure S6. Effect of modified gold nanoparticles (AuNPs) on polymerase chain reaction (PCR). The effect of modified AuNPs on PCR activity was determined by adding modified AuNPs to the PCR mixture at different ratios (w/w, 1, 2.5 and 10).


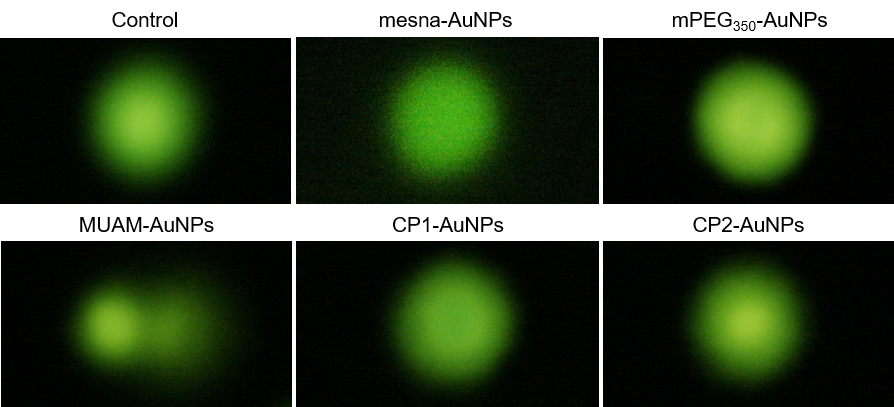


Supplementary Figure S7. DNA damage induced by gold nanoparticles (AuNP). The levels of AuNPs-induced DNA damage were examined by Comet assay using fluorescence microscopy. The presence of tail of the comet suggest the DNA damage in MUAM-AuNPs treated samples.


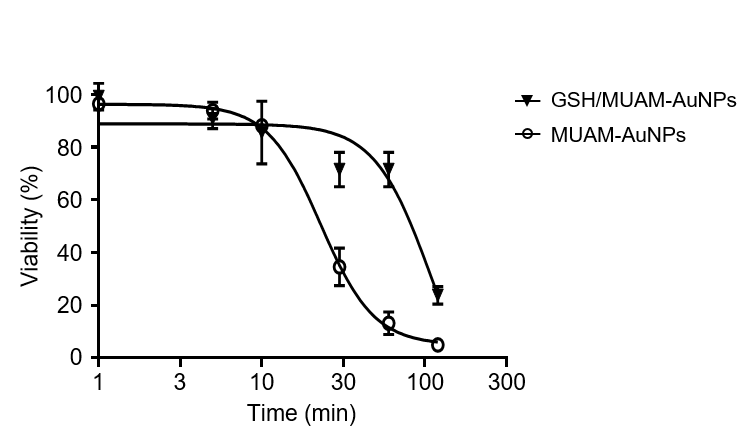


Supplementary Figure S8. Effect of a reducing agent to moderate gold nanoparticles (AuNPs)-induced cytotoxicity. HeLa cells were treated with 28 μg/ml MUAM-AuNPs with or without glutathione (1 mM, GSH) pre-treatment. Cell viability was then assessed by MTT assay in time-dependent manner. Pre-treatment with GSH delayed cell death of MUAM-AuNPs treated cells.


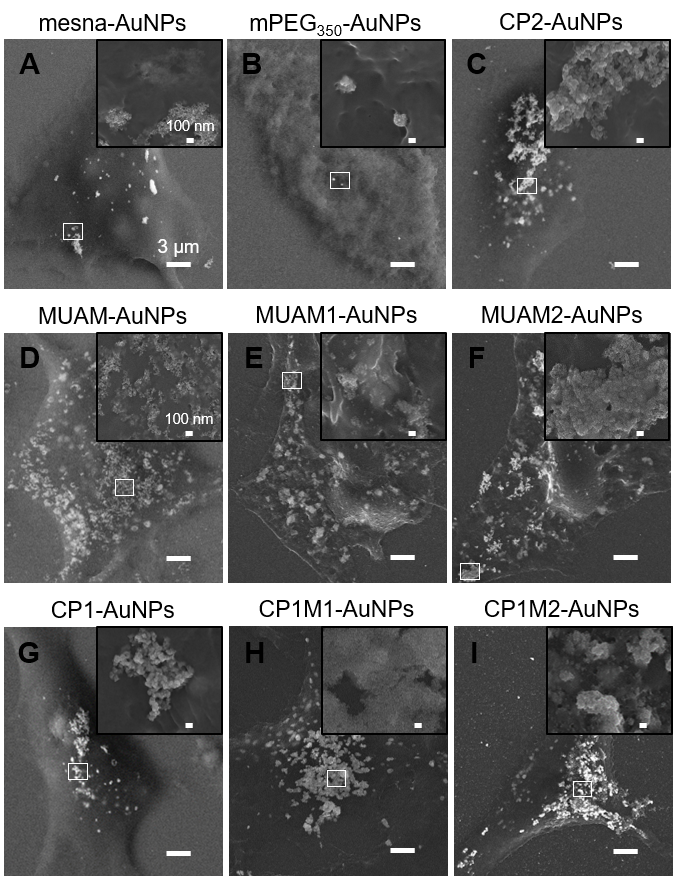


Supplementary Figure S9. FE-SEM image of modified gold nanoparticles (AuNPs)-treated HeLa cell. FE-SEM image are obtain after 24 h exposure to modified AuNPs, such as mesna- (A), mPEG_350_- (B), CP2- (C), MUAM- (D), MUAM1- (E), MUAM2- (F), CP1- (G), CP1M1- (H), and CP1M2-AuNPs (i). Scale bars: 3 μm. Higher magnification of FE-SEM images are shown (inset) with a 100 nm scale bar.


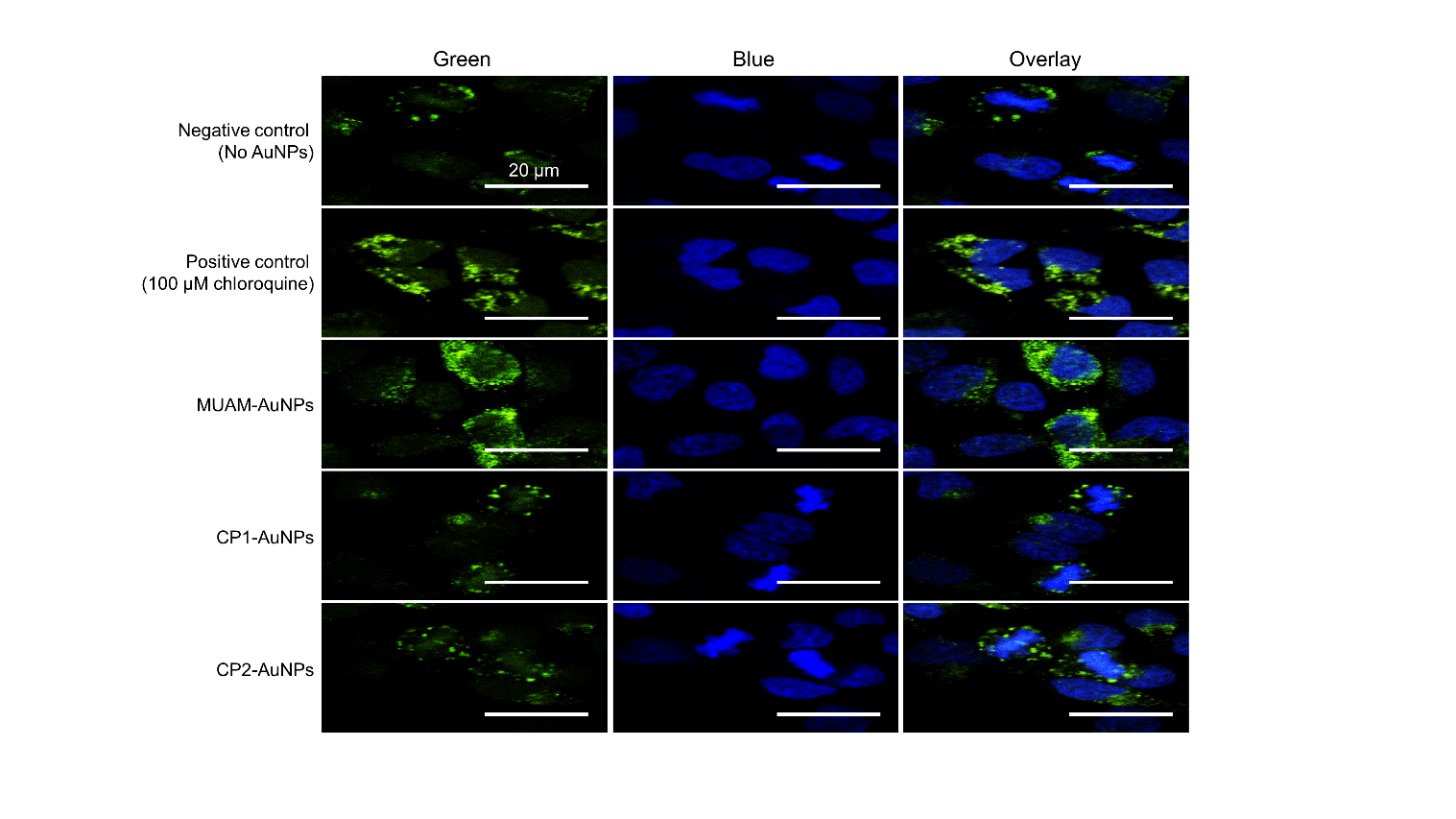


Supplementary Figure S10. Staining of mammalian cells for lysosomal integrity using Lucifer yellow. Cells were treated with MUAM-. CP1-, and CP2-AuNPs, individually. Not treated cells were included as a negative control and Chloroquine treated cells were used as a positive control. Only the MUAM-AuNPs treated cells showed cytoplasmic staining as the positive control cells, suggesting that the MUAM-AuNPs induces impairment of lysosomal membranes. scale bar: 20 µm


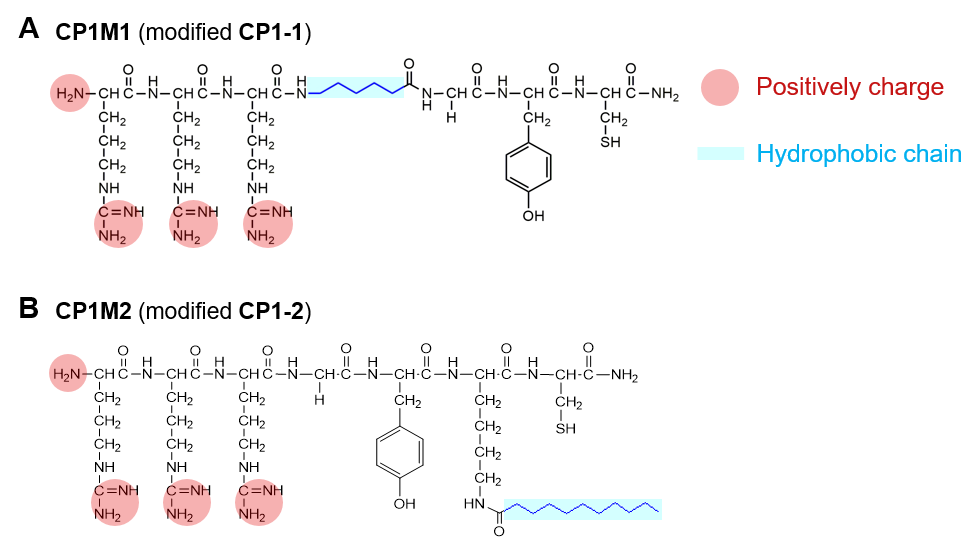


Supplementary Figure S11. Structure of CP1M1 (A) and CP1M2 (B).


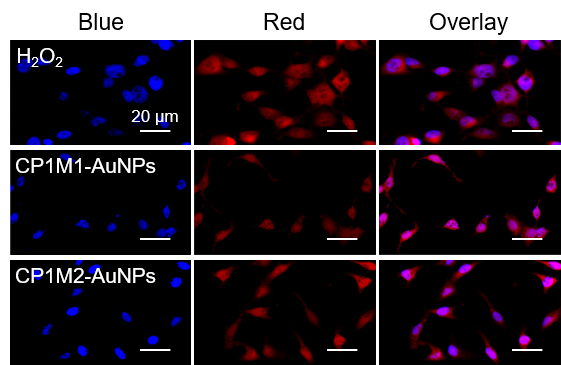


Supplementary Figure S12. Reactive oxygen species (ROS) overproduction induced by CP1M1- and CP1M2-AuNPs. HeLa cells were incubated with 200 μM H_2_O_2_, 20 μg/ml CP1M1-AuNPs, and 20 μg/ml CP1M2-AuNPs, respectively, and stained with Deep Red and DAPI. Red signal indicated intracellular ROS level.


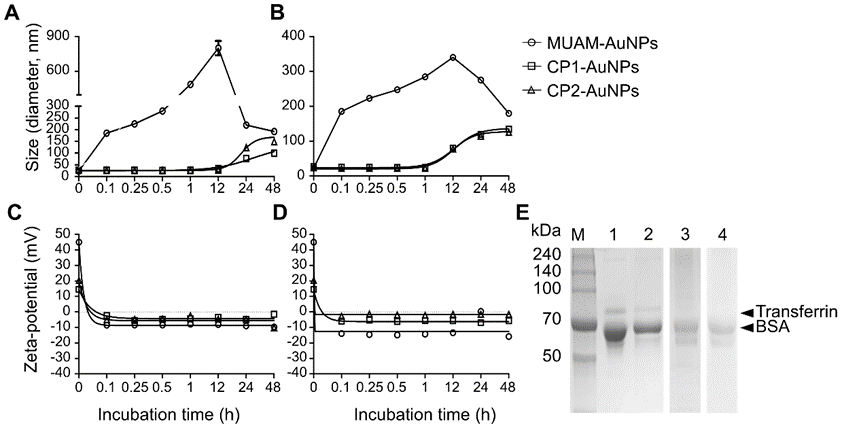


Supplementary Figure S13. Time dependent study on the protein corona formation of cationic gold nanoparticles (AuNPs) in biological medium. The size of AuNPs were measured using DLS in D1 (A) and D10 (B) media. The surface charge of cationic AuNPs were measured in D1 (C) and D10 (D) media. NP-bound proteins were analyzed by using gel electrophoresis (E). M, protein weight marker; Lane 1, D10 media; lane 2, MUAM-AuNPs; lane 3, CP1-AuNPs; lane 4, CP2-AuNPs. Lane 3 and 4 are acquired separately.


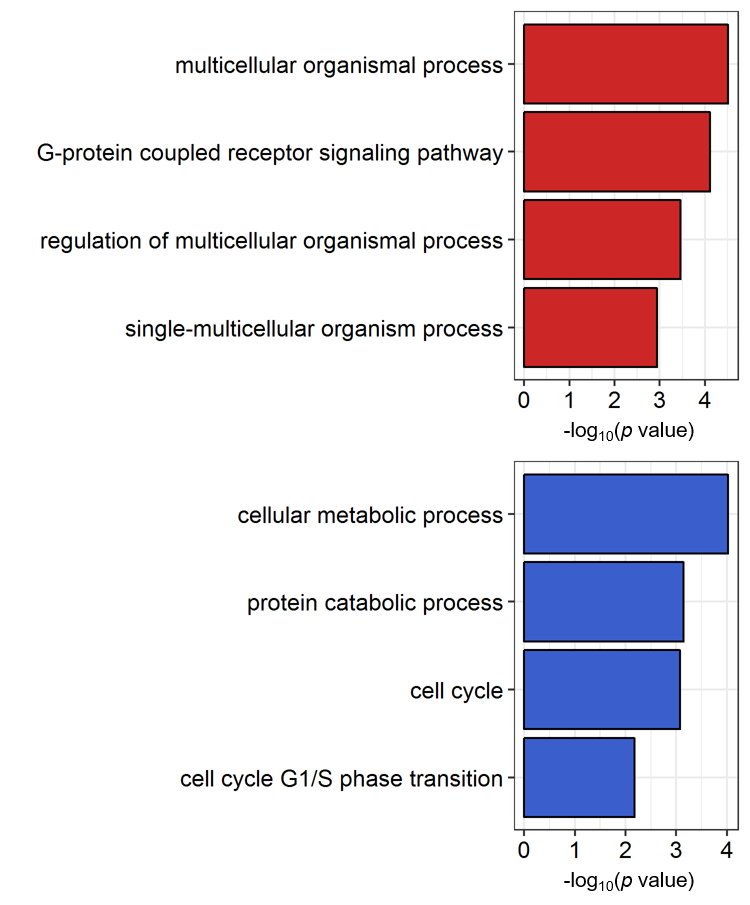


Supplementary Figure S14. Gene Ontology (GO) enrichment analysis of up- and down-regulated genes found in MUAM-AuNPs treated cells compared with other three samples (control, CP1- and CP2-AuNPs treated cells). (A) Top 4 significant GO terms found in upregulated genes (730 genes), (*p* < 0.05). (B) Top 4 significant GO terms found in downregulated genes (426 genes), (*p* < 0.05).

**
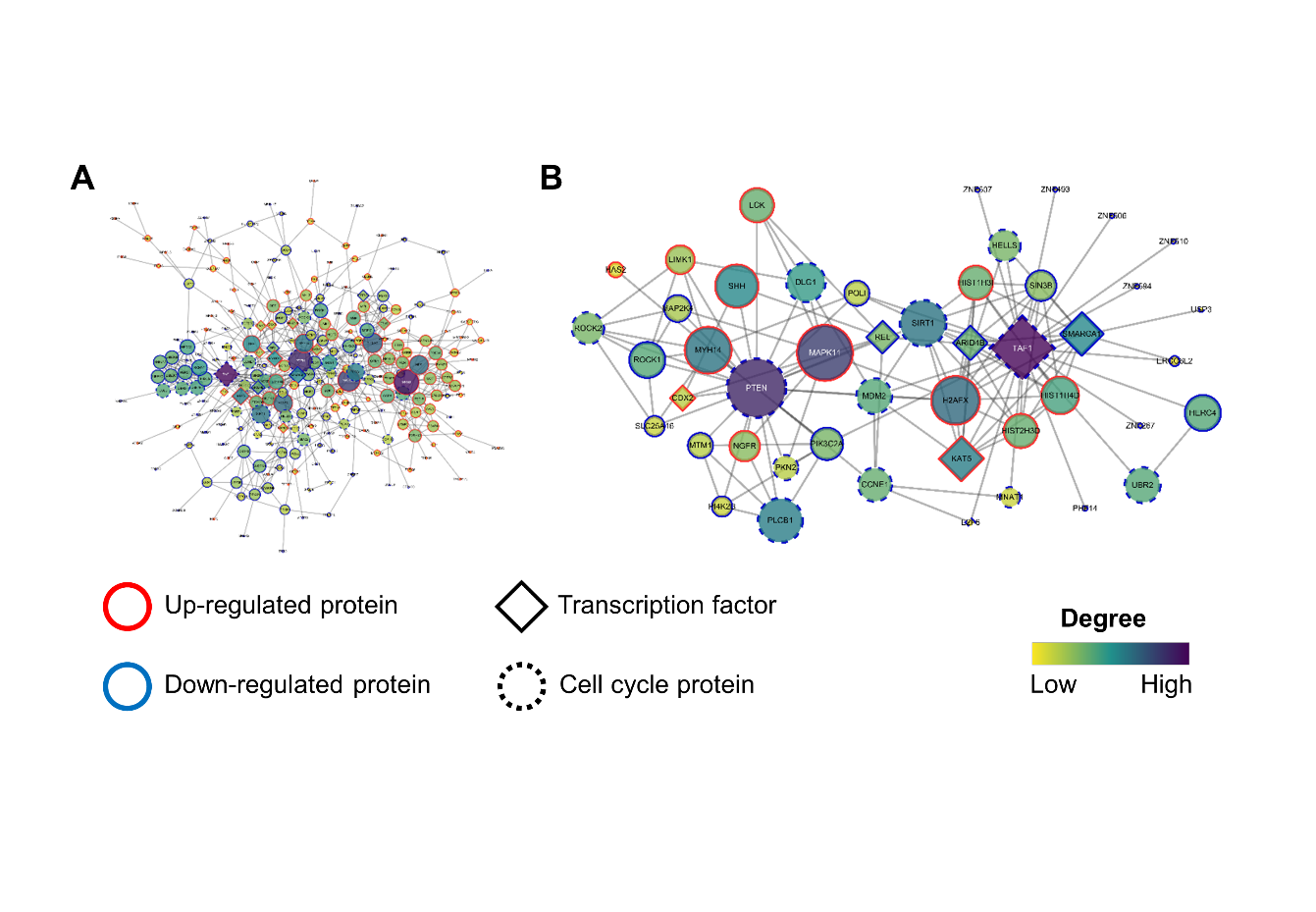
**

Supplementary Figure S15. The protein-protein interaction network of the DEGs involved in significant GO terms. (A) The whole network shows 249 nodes and 642 edges. (B) The subnetwork connected with hub proteins (TAF1 and PTEN) shows 47 nodes and 130 edges. Transcription factors are indicated as diamonds and other proteins are as circles. The node color represents degree (the neighborhood connectivity). The dotted border represents the proteins involved in the cell cycle (GO:0007049) or G1/S phase transition (GO:0044843) GO terms in Fig. S14.
